# Supplementary material for: Characterization of the COPD alveolar niche using single-cell RNA sequencing
Source: Nat Commun. 2022 Jan 25;13:494. doi: 10.1038/s41467-022-28062-9 (PMC8789871; doi:10.1038/s41467-022-28062-9)

SUPPLEMENTAL INFORMATION

Supplemental Table 1: Demographic Data.

Demographics and pulmonary function test results from control and COPD subjects. (n) = number of subjects with available data. * Summary statistics for pack-years in control subjects reflect current/former smokers only. For continuous variables, data reflects mean ± standard deviation).

	Control	COPD
Number of subjects	15	17
Sex		
female	8	8
male	7	9
Age	59.0 ± 10.5	62.6 ± 4.8
Ever smoker	4	17
Pack-years*	15± 8.7 (3)	53.5± 33.4 (16)
Lung Function		
FEV_1 (%)	–	21.0 ± 5.0 (15)
FVC (%)	–	48.2 ± 15.3 (15)
FEV_1/FVC	–	0.36 ± 0.05 (14)
TLC (%)	–	122.8 ± 24.4 (9)
FRC (%)	–	161.2 ± 57.2 (11)
D_{LCO} (%)	–	20.4 ± 8.2 (13)

Supplemental Table 2: Cells, differential expressed genes, and marker genes per cell type.
Total number of cells analyzed per cell type and the number of differentially expressed genes between COPD and control (two-sided Wilcoxon rank sum, FDR <0.05). Marker genes for each cell type are shown.

Cell	# Differentially Expressed Genes	# Cells Control	# Cells COPD	Marker genes
AT1	69	268	363	*AGER, EMP2*
$AT2_S$	29	313	378	*SFTPC, TNIK, ERBB4, ANK3, TACC2*
$AT2_B$	217	293	405	*SFTPC, SFTPA1, HHIP*
Aberrant Basaloid	–	0	33	*KRT17, MMP7, TP63*
Basal	3	52	89	*KRT5, MIR205HG*
Ciliated	171	544	810	*FOXJ1, EPCAM*
Club	13	123	444	*SCGB3A1, SCGB1A1*
Goblet	5	59	117	*MUC1, BPIFB1*
Pulmonary neuroendocrine cell	0	15	12	*CHGA, GRP*
Fibroblast Adventitial	235	544	336	*PLA2G2A, COL1A2*
Fibroblast Alveolar	88	159	349	*MOXD1, COL1A2*
Fibroblast CTHRC1+	0	8	63	*COL1A2, ACTA2*
Pericytes	1	23	67	*COX4I2, KCNK3*
Smooth Muscle Cells	6	64	79	*DES, PLN*
Mesothelial	13	34	280	*WT1, MSLN, HAS1*
Lymphatic	93	615	366	*TFF3, LYVE1*
gCap	59	182	121	*CA4, VWF,*
Aerocyte	82	395	298	*CA4, HPGD, EDNRB*
Arterial	43	179	139	*IGFBP3, VWF*
Venous	4	108	64	*HDAC9, VWF*
Peribronchial	2	52	73	*COL15A1, VWF*
Classical Monocyte	237	4050	3759	*S100A12, VCAN*
Nonclassical Monocyte	226	851	2141	*LILRB2, LILRA5*
Interstitial Macrophage	191	16008	6990	*FPR3, MRC1*
Macrophage Alveolar	151	15107	22221	*PPIC, AMIGO2, MRC1*
Conventional Dendritic Cell 1	226	727	1416	*CADM1, CLEC9A, WDFY4*
Conventional Dendritic Cell 2	48	140	508	*CLEC10A, TMEM163*
Dendritic Cell Mature	33	82	223	*LAMP3, CCR7*
Dendritic Cell Langerhans	0	7	14	*S100B, CD1A, FCER1A*
Plasmacytoid Dendritic Cell	18	42	209	*GMB, VASH2*
Mast	14	127	648	*TPSAB1, MS4A2, CPA3*
B	41	650	2159	*MS4A1, BCL11A, CD79A*
B Plasma	20	135	696	*DERL3, CD79A, IGHG1*
T	53	3089	5772	*GPR171, THEMIS, CD4*
T Cytotoxic	56	2462	5469	*CCL5, CD8A*
T Regulatory	6	196	235	*FOXP3, CTLA4*
Natural Killer Cell	43	2007	3929	*NKG7, GZMB, S1PR5*
Innate Lymphoid Cell A	12	192	172	*AREG, MCTP2*
Innate Lymphoid Cell B	3	73	117	*AREG, FXYD5*

Supplemental Table 3: CXCL ligand-receptor pairs. CXCL ligand-receptor pairs in the Fantom5 database.

Ligand	Receptor
CXCL1	CXCR1
CXCL1	CXCR2
CXCL1	DARC
CXCL10	CXCR3
CXCL10	SDC4
CXCL11	ACKR3
CXCL11	CXCR3
CXCL12	CD4
CXCL12	CXCR3
CXCL12	SDC4
CXCL12	ACKR3
CXCL12	CXCR4
CXCL12	ITGB1
CXCL13	HTR2A
CXCL13	OPRD1
CXCL13	ACKR4
CXCL13	CXCR3
CXCL13	CXCR5
CXCL16	CXCR6
CXCL2	CXCR1
CXCL2	CXCR2
CXCL2	XCR1
CXCL3	CXCR1
CXCL3	CXCR2
CXCL5	CXCR1
CXCL5	CXCR2
CXCL5	DARC
CXCL6	CXCR1
CXCL6	CXCR2
CXCL9	CXCR3
PF4	CXCR3
PF4	LDLR
PF4	PROCR
PF4	SDC2

Supplemental Figure 1: Mouse single-cell RNAseq. A) Uniform Manifold Approximation and Projection (UMAP) representation of 19,311 cells from 4 cigarette smoke (CS)-exposed mice (2 male and 2 female) and 20,410 cells from only room air (RA)-exposed mice (2 male and 2 female), grouped into 35 distinct cell types (*left*) with identification of CS-exposed (red) and RA-exposed cells (blue) (*right*). **B)** Heatmap of z-scores of marker gene expression values, grouped broadly into epithelial, endothelial, and stromal cell types. Each column represents expression values for an individual cell. Columns are hierarchically ordered by cell type, exposure to RA or CS, and then by individual mouse.

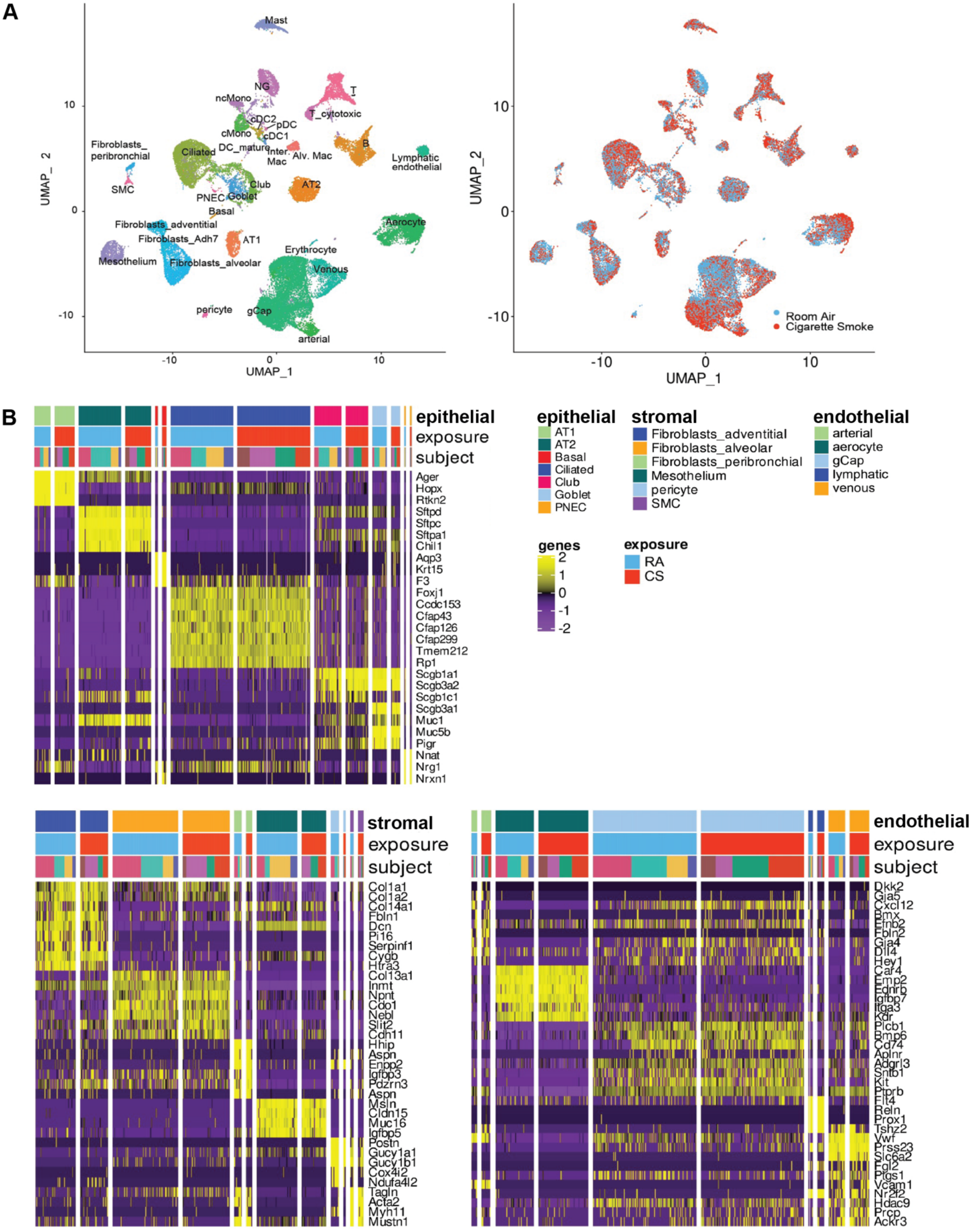

Supplemental Figure 2: Quantifying disease severity in mice exposed to cigarette smoke. A) Mean linear intercept measured in mice exposed to 10 months of cigarette smoke (CS) or room air (RA) (n=4/group). *P = 0.03, two-sided Wilcoxon rank sum. **B)** Concentration of $CD45^{+}$ cells in lung tissue lysates from mice exposed to 10 months of RA or CS. Bar represents median (**A,B**).

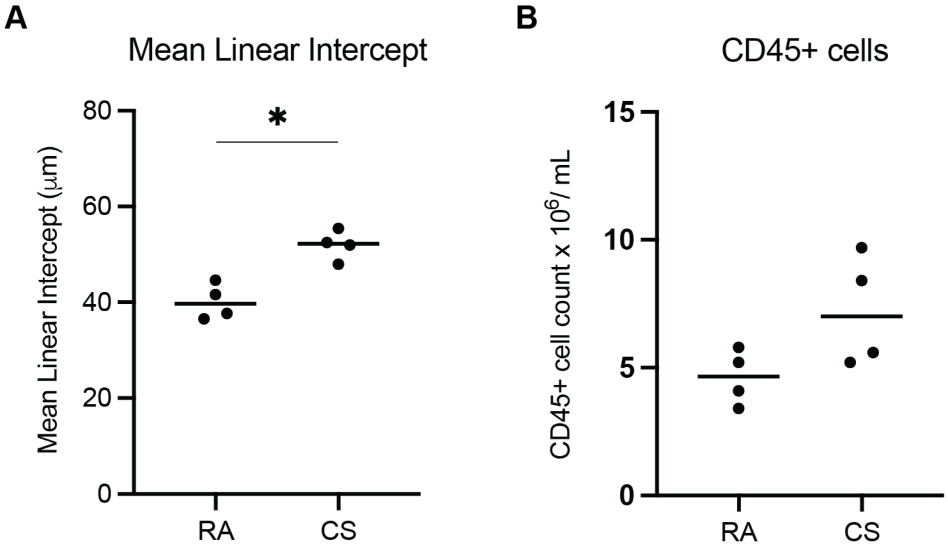

Supplemental Figure 3: Feature plot of $AT2_S$ and $AT2_B$ marker genes. A) Uniform Manifold Approximation and Projections (UMAPs) of AT1, $AT2_S$ and $AT2_B$ cells. Brown = AT1 Cells, Blue = $AT2_S$ cells, and Green = $AT2_B$ cells. **B)** Feature plots of normalized marker gene expression in $AT2_S$ (*TNIK, ERBB4*) vs. $AT2_B$ cells (*SFTPA1, SFTPC, HHIP, WIF1*).

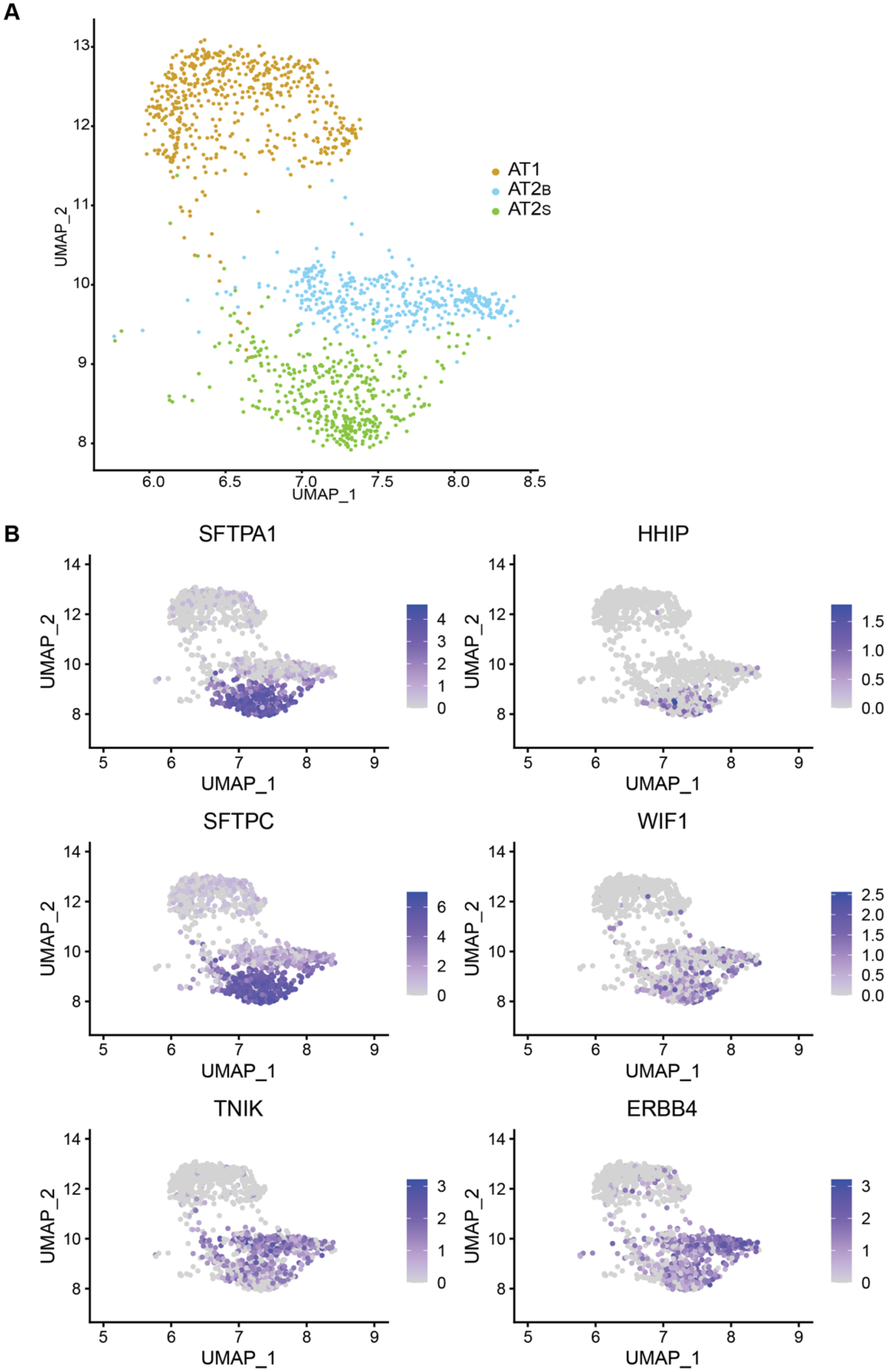

Supplemental Figure 4: *HHIP* expression per cell type. Number of *HHIP* transcripts detected per cell type.

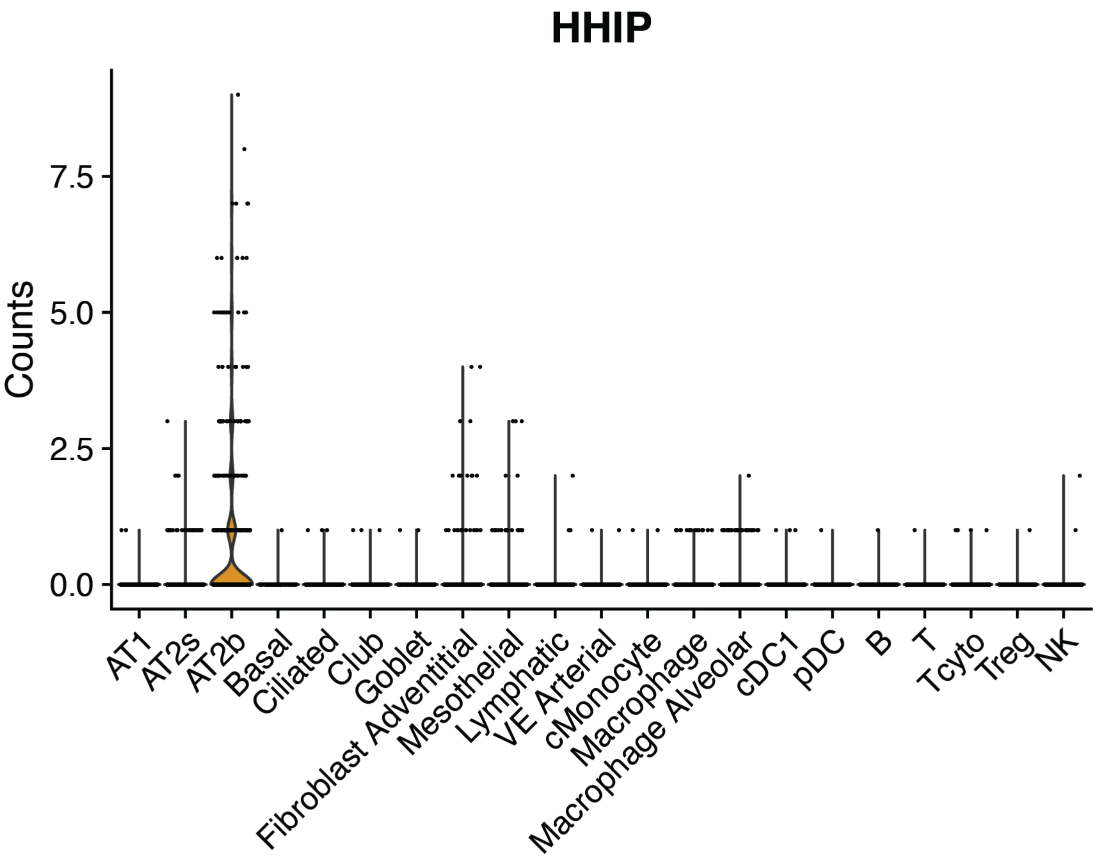

Supplemental Figure 5: Co-localization of *HHIP* and *SFTPA1* in AT2 cells. Immunofluorescence staining for pro-surfactant protein C (SFTPC) (purple) *in situ* hybridization for *HHIP* mRNA (red), *SFTPA1* mRNA (green), and DAPI (blue) in normal human lung tissue samples. Bar = 100 μm. Original magnification ×20. Inset shows a SFTPC^{+}, *HHIP*$^{+}$, *SFTPA1*$^{+}$ cell. Yellow arrows point to examples of SFTPC^{+}, *HHIP*$^{-}$, *SFTPA1*$^{-}$ cells. Images representative of 5 samples.

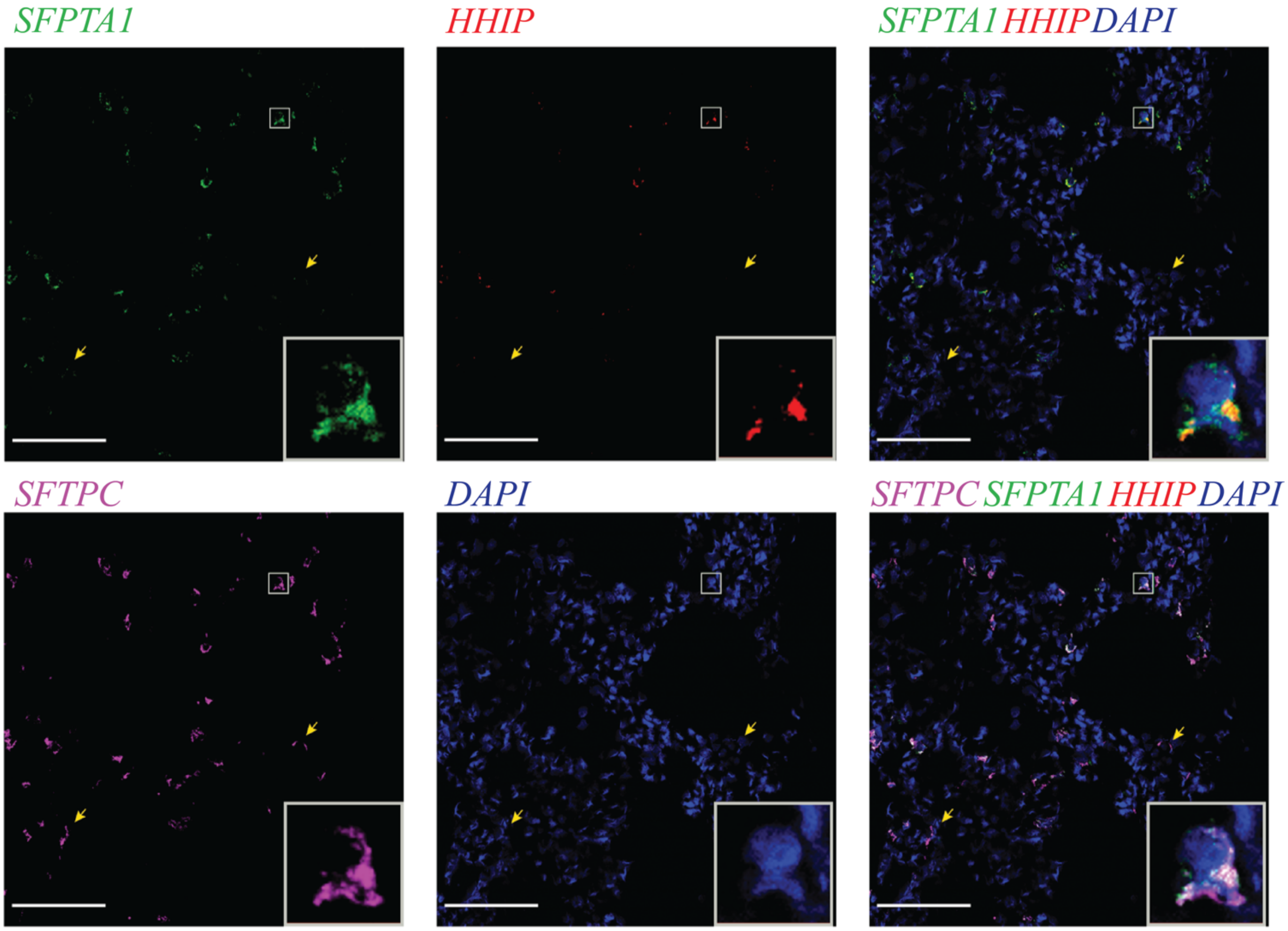

Supplemental Figure 6: Dot plot of z-scores for commonly associated GWAS genes. Dot size reflects percentage of cells with gene expression; color corresponds to degree of expression.

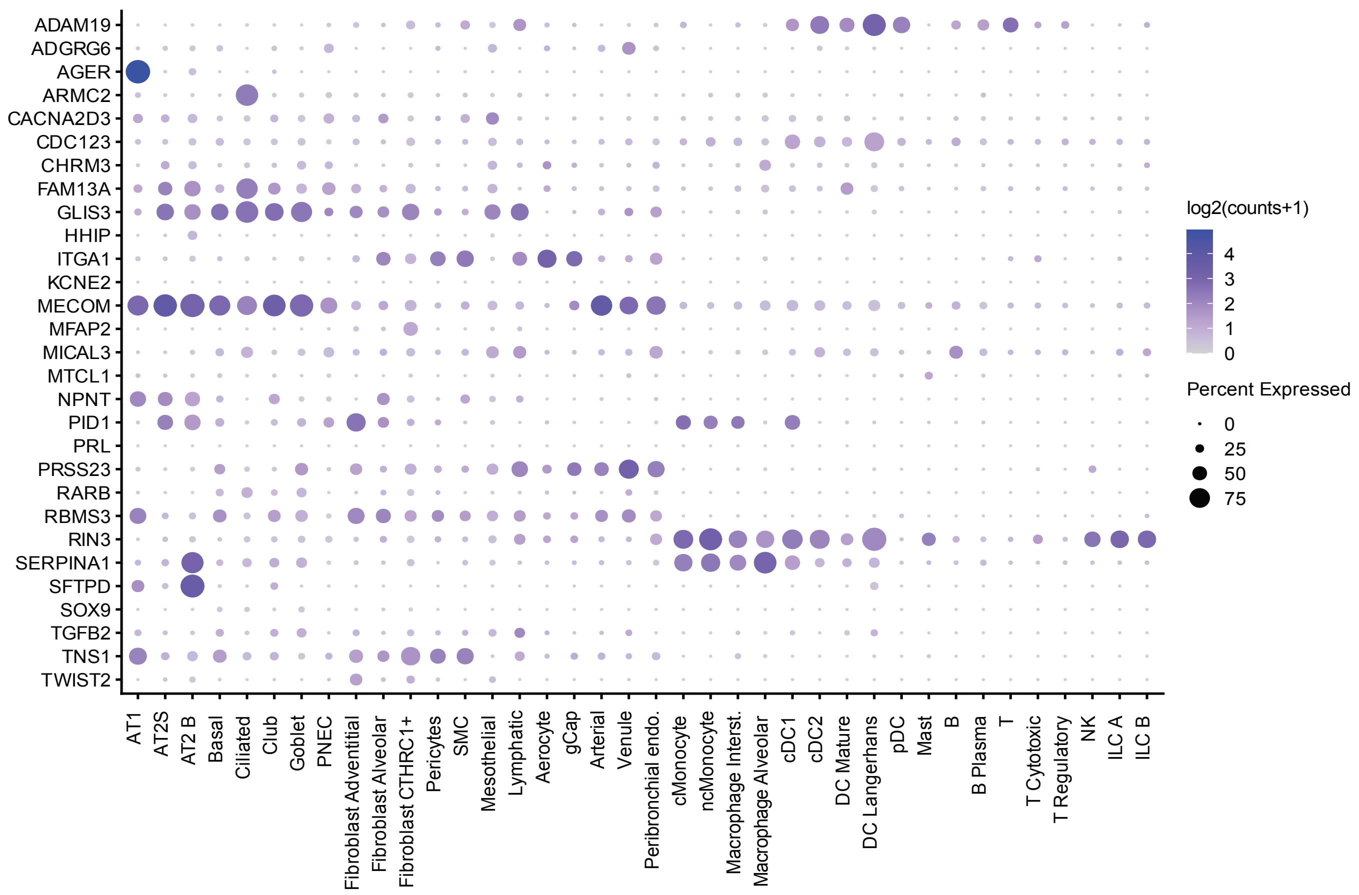

Supplemental Figure 7: A) Violin plot of normalized *CD74* expression in $AT2_B$ cells from non-smokers without COPD (n=11), former/current smokers without COPD (n=4), and former smokers with COPD (n=17) ***P=1.09x10^{-08} (two-sided Wilcoxon rank sum with Bonferroni correction). **B)** Violin plot of normalized *Cd74* expression in AT2 cells from mice exposed to cigarette smoke for 10 months (n=4) vs. room air (n=4) ***P = 3.72x10^{-21}, (two-sided Wilcoxon rank sum with Bonferroni correction).

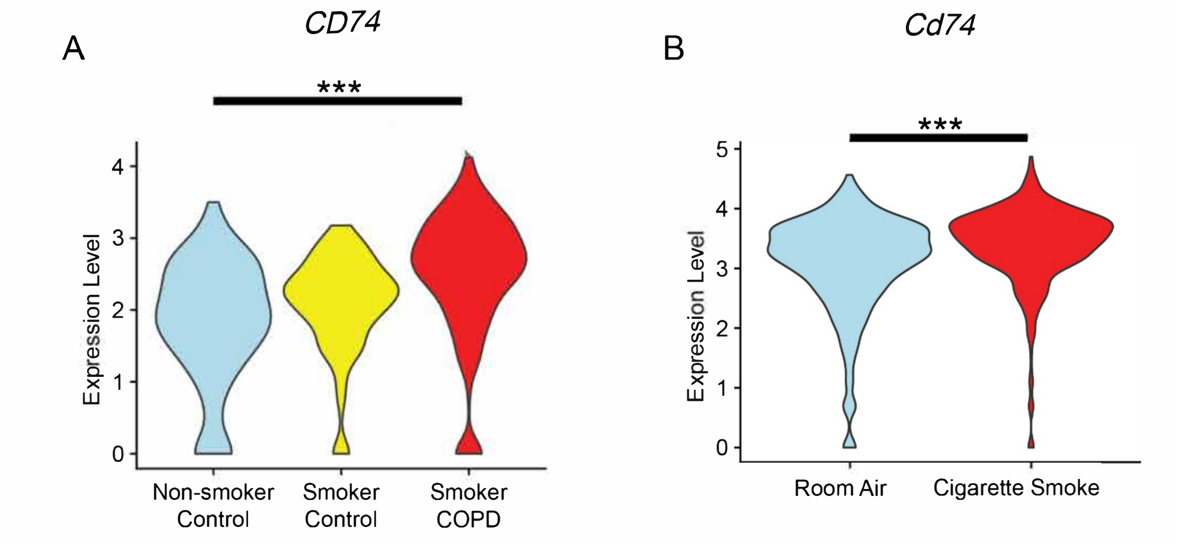

Supplemental Figure 8: NUPR1 in AT2 cells. Sample immunofluorescence staining for pro-surfactant protein C (SFTPC) (green), NUPR1(red), and DAPI (blue) in normal and COPD human lung tissue samples. Bar = 100 μm. Original magnification, ×20. Images representative of 5 control and 5 COPD samples.

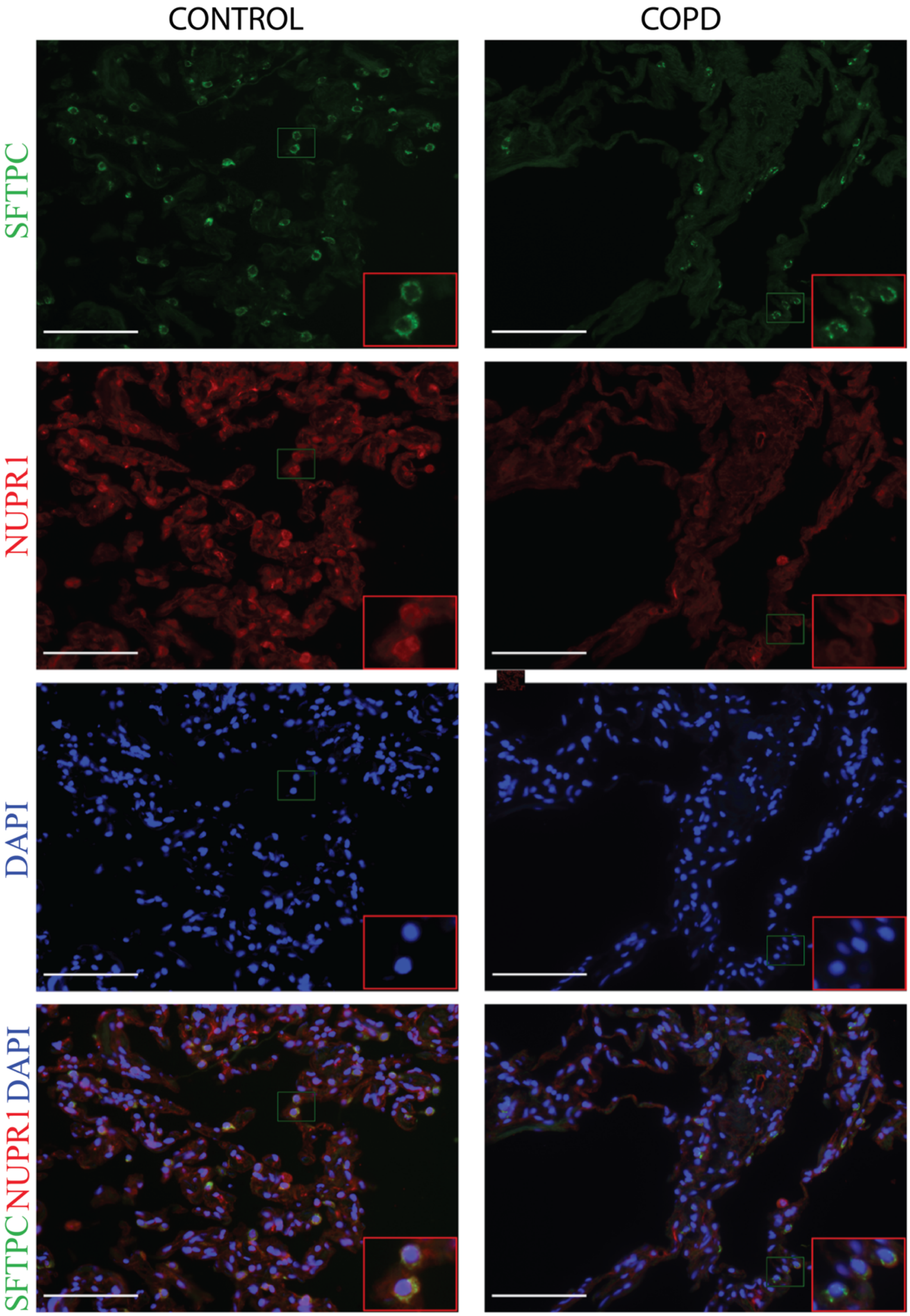

Supplemental Figure 9: *NUPR1* expression following exposure to cigarette smoke extract. *NUPR1* expression in A549 cells following treatment with 8% cigarette smoke extract. n=5/group at 0 hours, and 3/group at 8, 16, and 24 hours. Error bars represent median ± interquartile (IQR) range.

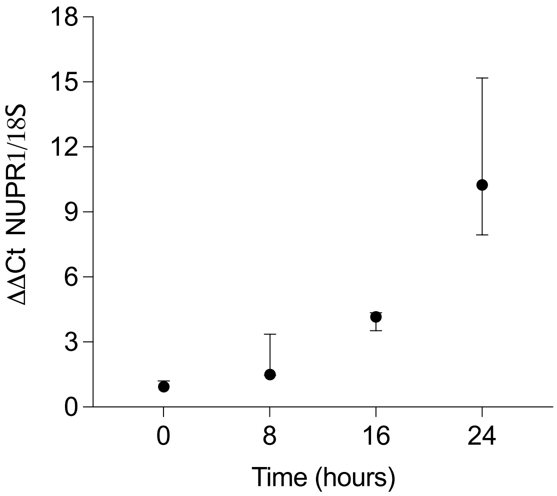

Supplemental Figure 10: Silencing RNA against *NUPR1*. Relative *NUPR1* expression following treatment with silencing RNA targeted against *NUPR1* mRNA (siNUPR1) or scrambled control (siCTRL) in **A)** A549 cells (n=4/group) *P=0.03 (two-sided Wilcoxon rank sum test). **B)** small airway epithelial cells (SAEC*) (*n=4/group*)* *P = 0.03 (two-sided Wilcoxon rank sum test). **C***)* Induced pluripotent stem cells (iPSC) - derived alveolar type 2 (AT2) cells grown at air-liquid interface (n=3/group). Error bars represent median ± interquartile range.

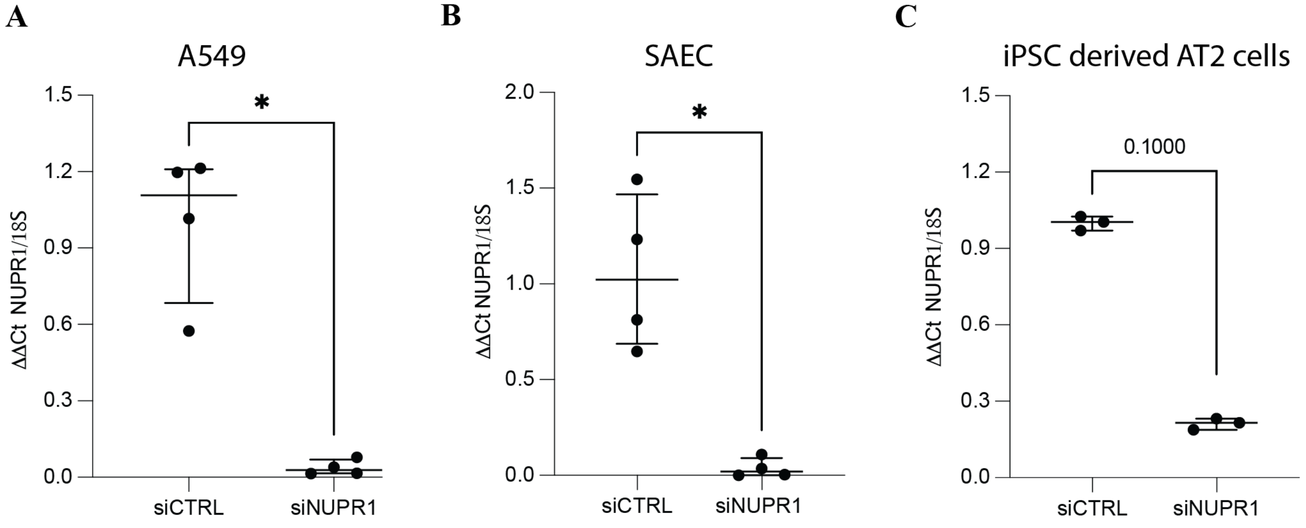

Supplemental Figure 11. Flow Cytometric detection of Annexin V and propidium iodide in A549 cells exposed to cigarette smoke extract. A) Flow cytometric detection of Annexin V-FITC (*x-axis*) and propidium iodide (*y-axis*) in A549 cells exposed to 0% cigarette smoke extract (CSE) or 12.5% CSE, treated with silencing RNA targeted against *NUPR1* mRNA (siNUPR1) vs. scrambled control (siCTRL), and treated with deferoxamine mesylate (DEF) (100 µM) vs. vehicle control. **B)** Single-stain (SS) controls are shown.

A

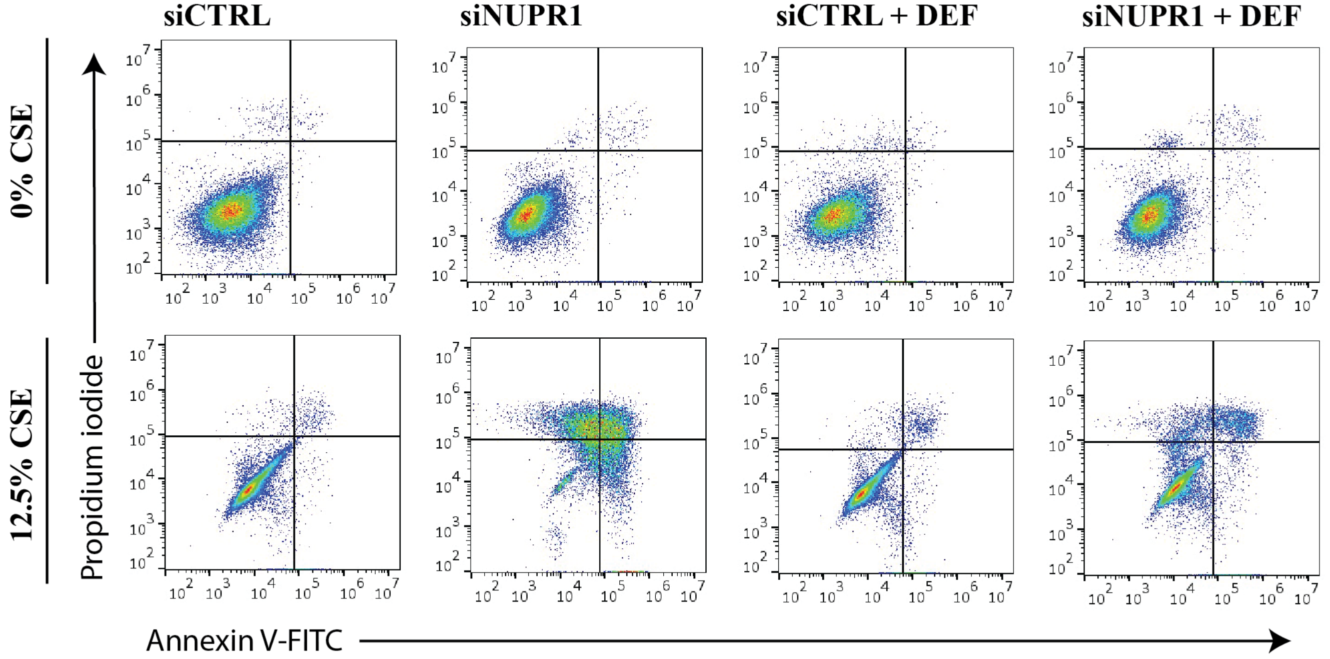

B

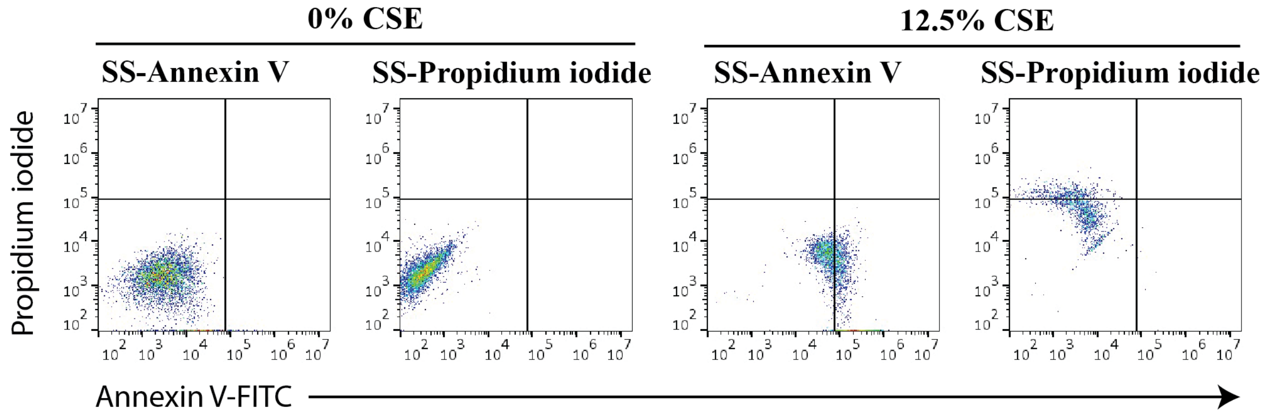

Supplemental Figure 12. Flow cytometric detection of Annexin V and propidium iodide in small airway epithelial cells exposed to cigarette smoke extract. A) Flow cytometric detection of Annexin V-FITC (*x-axis*) and propidium iodide (*y-axis*) in primary small airway epithelial cells exposed to 0% cigarette smoke extract (CSE) or 12.5% CSE, treated with silencing RNA targeted against *NUPR1* mRNA (siNUPR1) or scrambled control (siCTRL). **B)** Single-stain (SS) controls are shown.

A

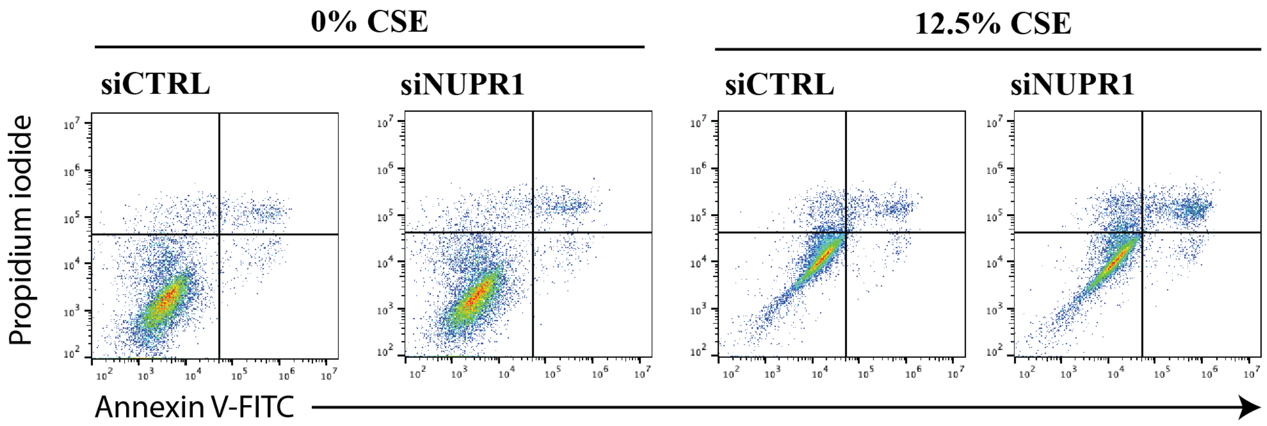

B

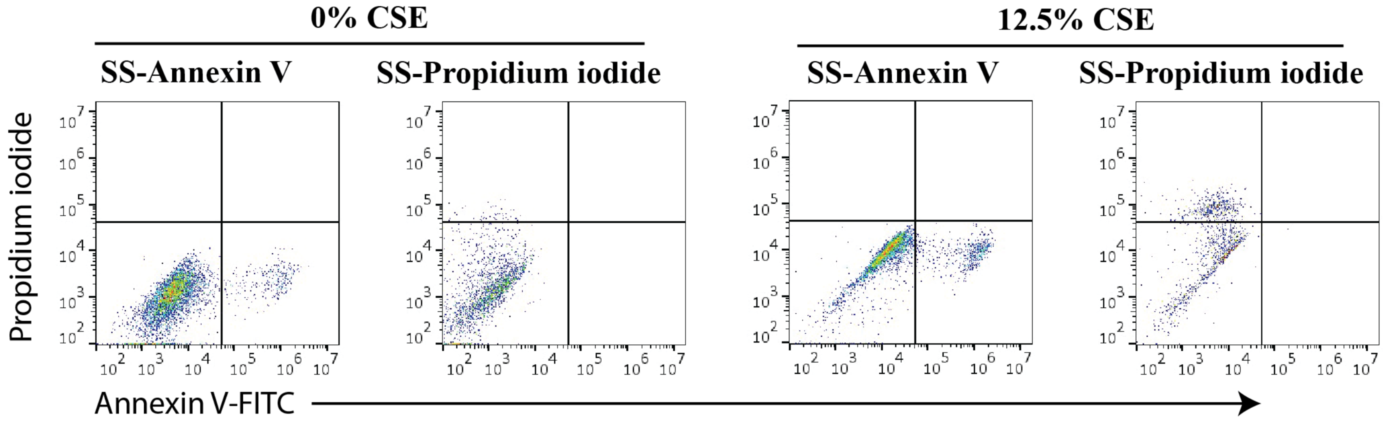

Supplemental Figure 13. Flow cytometric detection of caspase 3/7 in A549 cells exposed to cigarette smoke extract. A) Flow cytometric detection of Sytox AAdvanced (*y-axis*) and caspase 3/7-GFP (*x-axis*) in A549 cells exposed to 0% cigarette smoke extract (CSE) or 8% CSE and treated with silencing RNA targeted against *NUPR1* mRNA (siNUPR1) or scrambled control (siCTRL). **B)** Single-stain (SS) controls are shown. **C)** Box and whiskers plot of caspase 3/7-GFP and Sytox AAdvanced staining in A549 cells treated with silencing RNA targeted against *NUPR1* mRNA (siNUPR1) or scrambled control (siCTRL) exposed to 0% and 8% CSE. n=6/group. Boxes represent median and interquartile ranges (IQRs), whiskers include all points. **P<0.01 and ***P <0.001 using two-way ANOVA with Tukey post-hoc test.

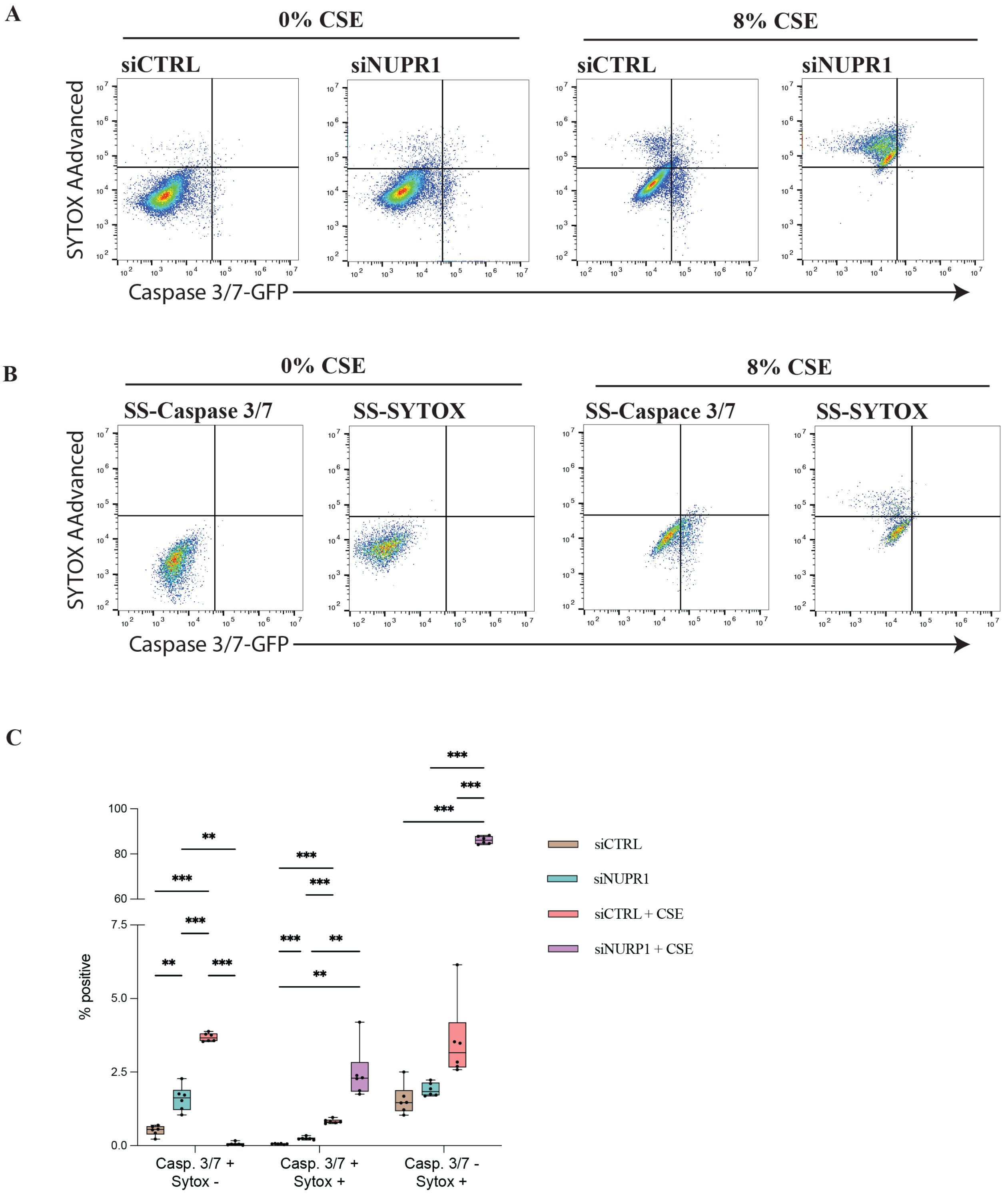

Supplemental Figure 14. Cytotoxicity and viability assays using regulated cell death inhibitors. A,B) Box and whisker plots of **A)** lactate dehydrogenase (LDH) assay and **B**) MTT assay of A549 cells treated with silencing RNA against *NUPR1* mRNA (siNUPR1) or scrambled RNA control in the absence or presence of 8% cigarette smoke extract (CSE) and treated with deferoxamine mesylate (100 µM), Z-VAD-FMK (50 µM), or necrosulfonamide (2 µM) for 24 h. $n = 17$/group for the LDH assay and 18/group for the MTT assay. Boxes represent median and interquartile ranges (IQRs), whiskers include all points. P-values generated using a two-way ANOVA with a Tukey post-hoc test are shown.

A

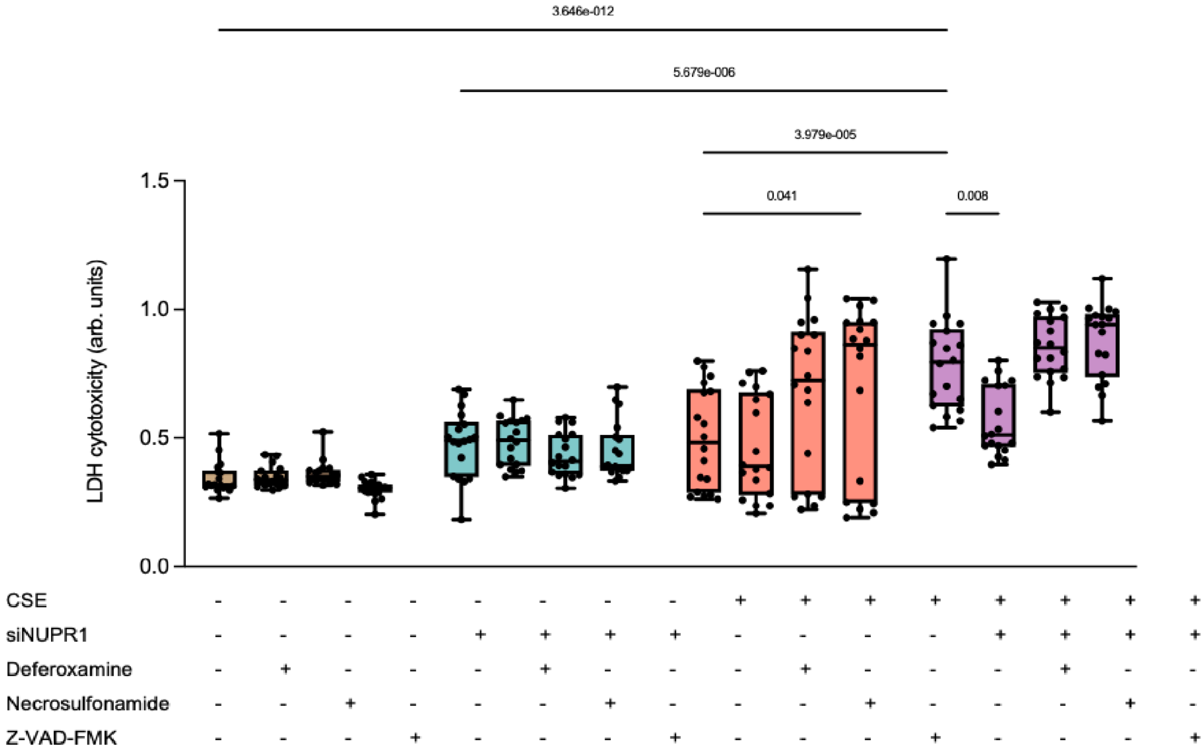

B

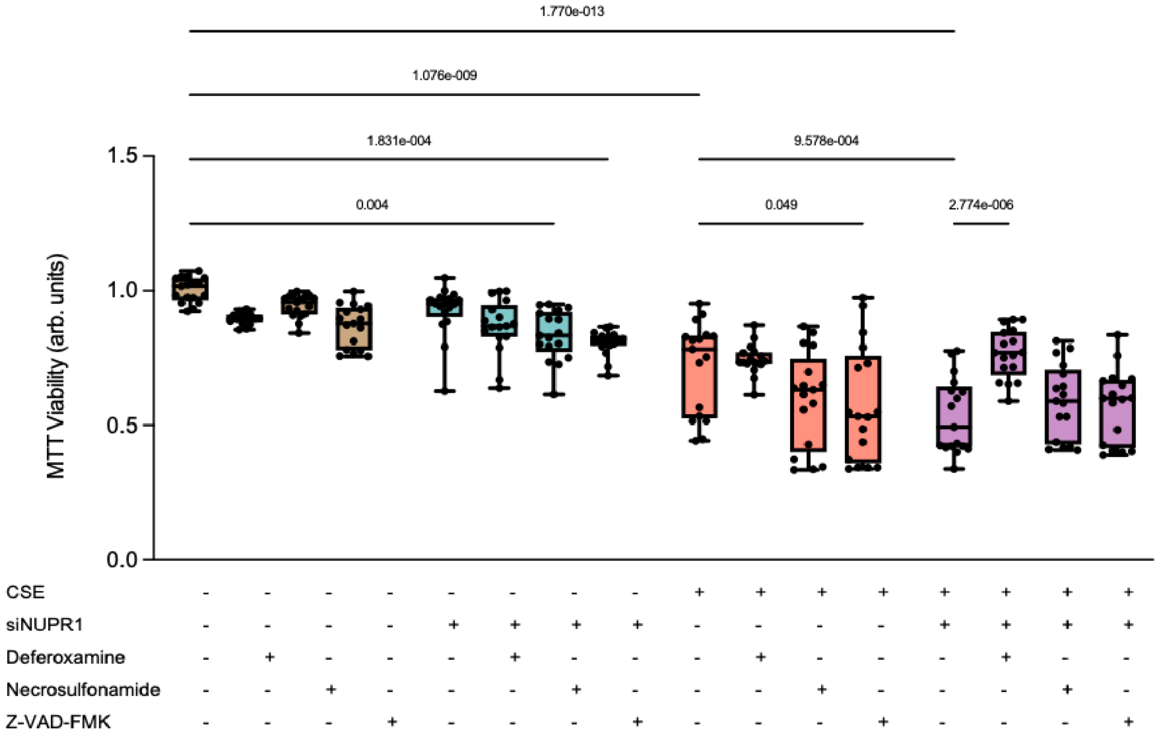

Supplemental Figure 15: *PRX* and *NOSTRIN* expression. Normalized expression level of **A**) *PRX* and **B)** *NOSTRIN* by cell type.

A

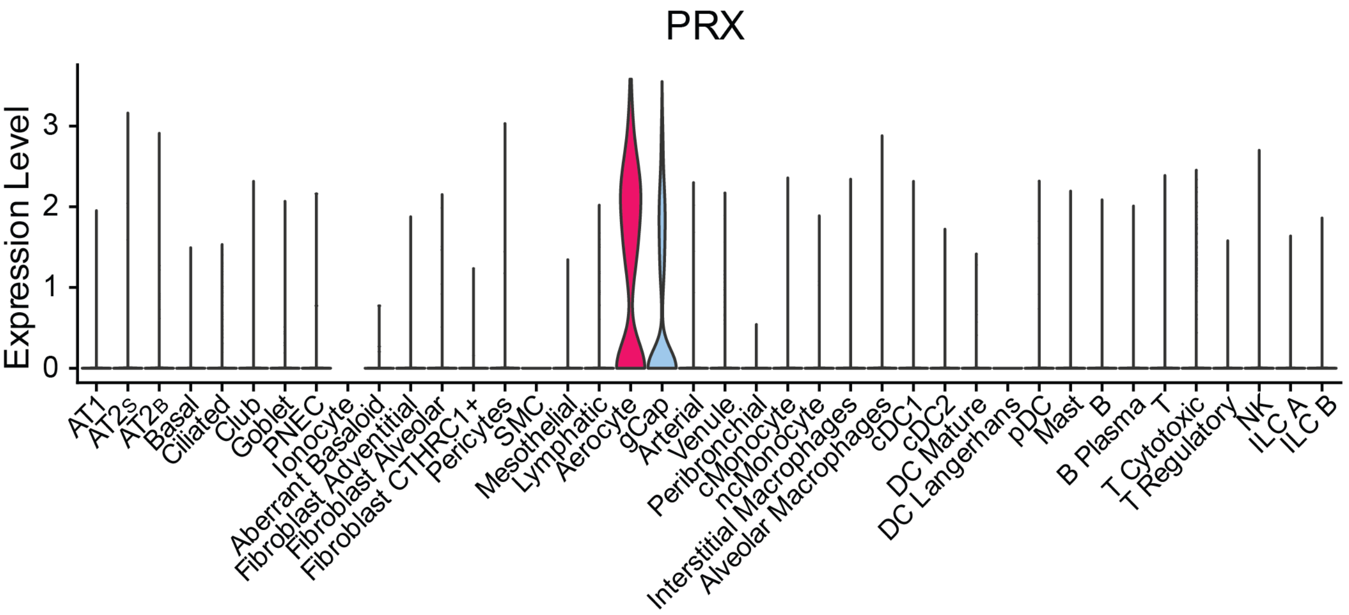

B

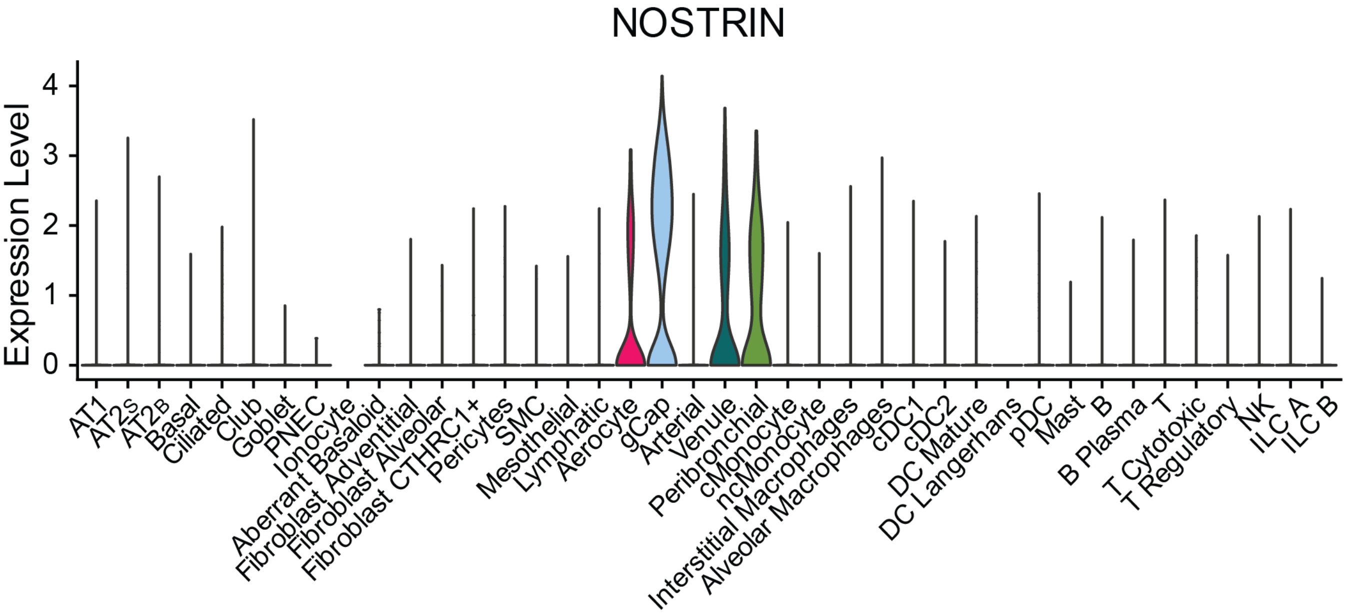

Supplemental Figure 16: Cell proportion makeup of endothelial and stromal cells.
A,B) Boxplots of cellular proportions present in COPD and control lung tissues based on fraction of the number of cells per subject for **A**) endothelial cells and **B**) stromal cells within the single cell dataset (n = 17 COPD subjects and 15 control subjects). Boxes represent median and interquartile ranges (IQRs); whiskers are 1.5 x IQR, and dots represent subjects outside the IQR range. SMC = smooth muscle cells, gCap = general capillary cells.

A

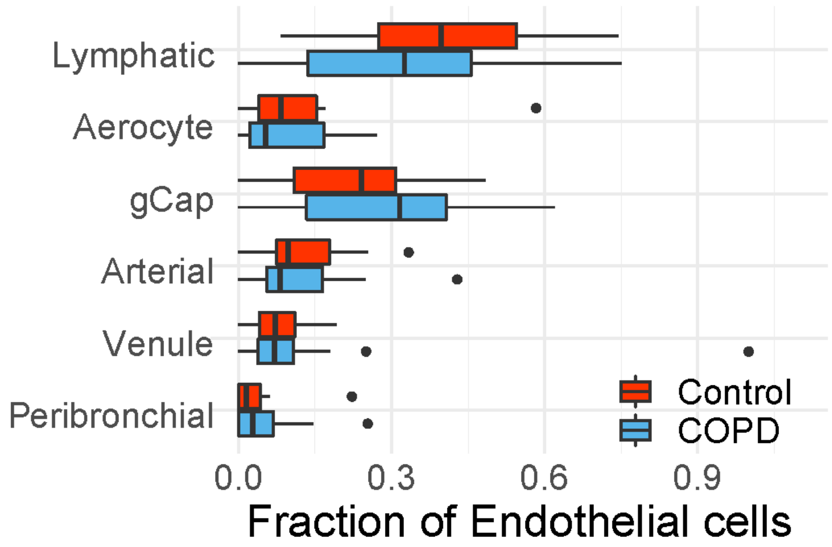

B

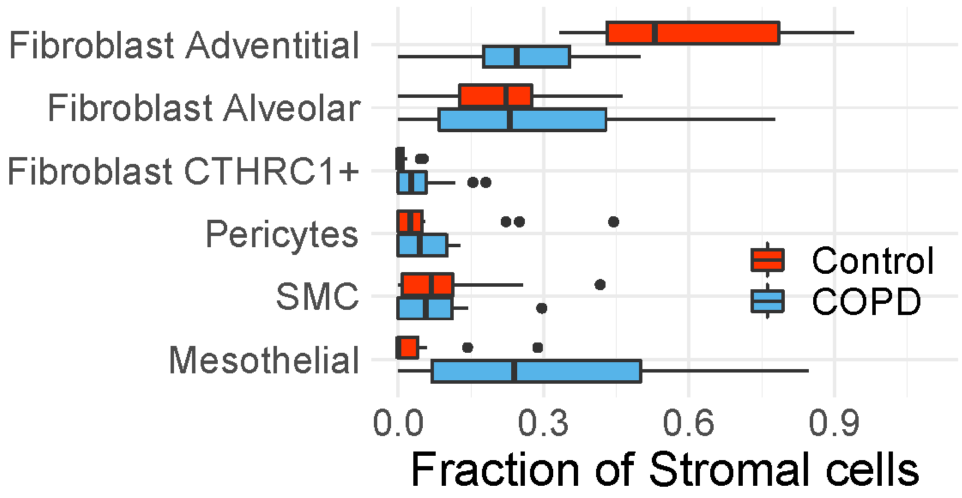

Supplemental Figure 17: Differential circos plots demonstrating changes in $AT2_S$ and $AT2_B$ signaling. A) Outgoing AT_S connectome, **B)** Incoming AT_S connectome **C)** Outgoing $AT2_B$ connectome, **D)** Incoming $AT2_S$ connectome. AT1 = alveolar epithelial type I cells, AT2 = alveolar epithelial type II cells, PNEC = pulmonary neuroendocrine cells, SMC = smooth muscle cells, gCap = general capillary, cMonocyte = classical monocytes, ncMonocyte = non-classical monocyte, Macs. = macrophages, DC = dendritic cells, cDC = conventional dendritic cells, pDC = plasmacytoid dendritic cells, NK = natural killer cells, ILC = innate lymphoid cells, Fibro = fibroblast.

A

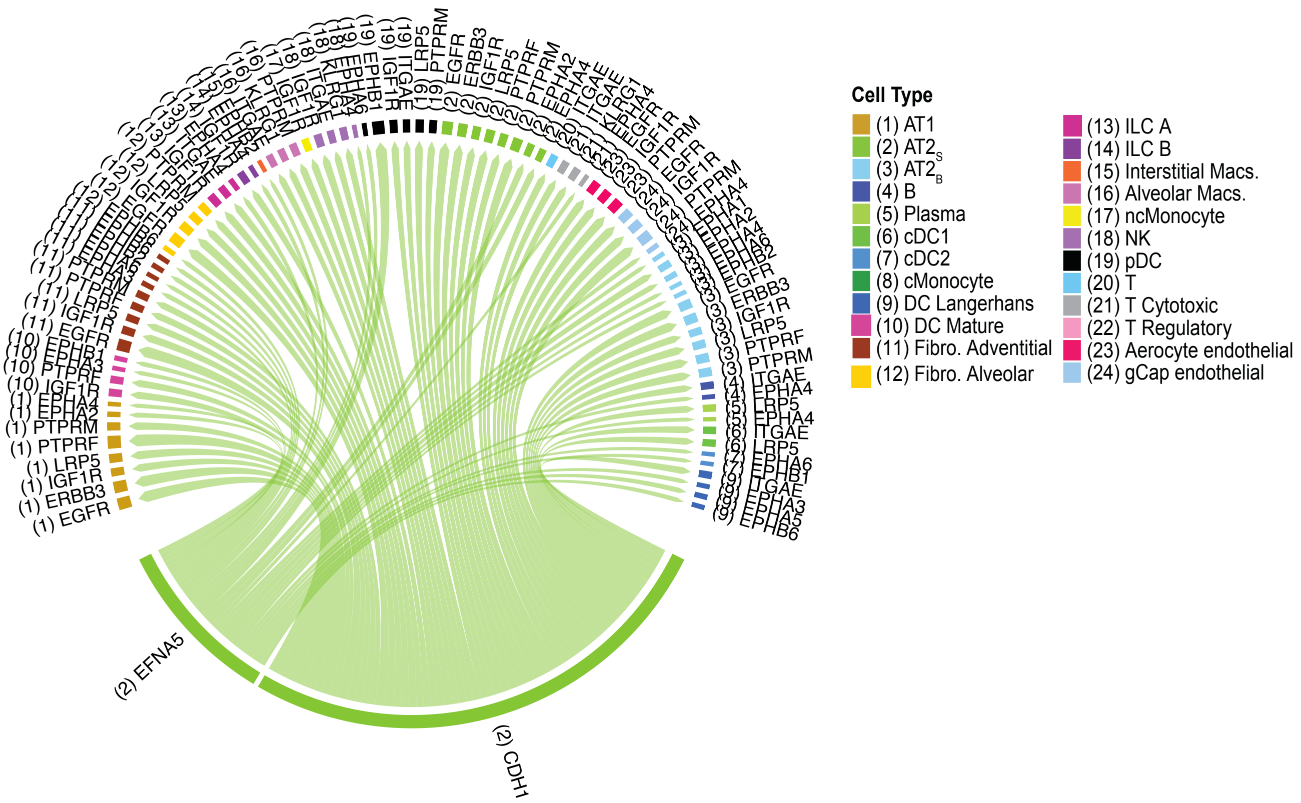

B

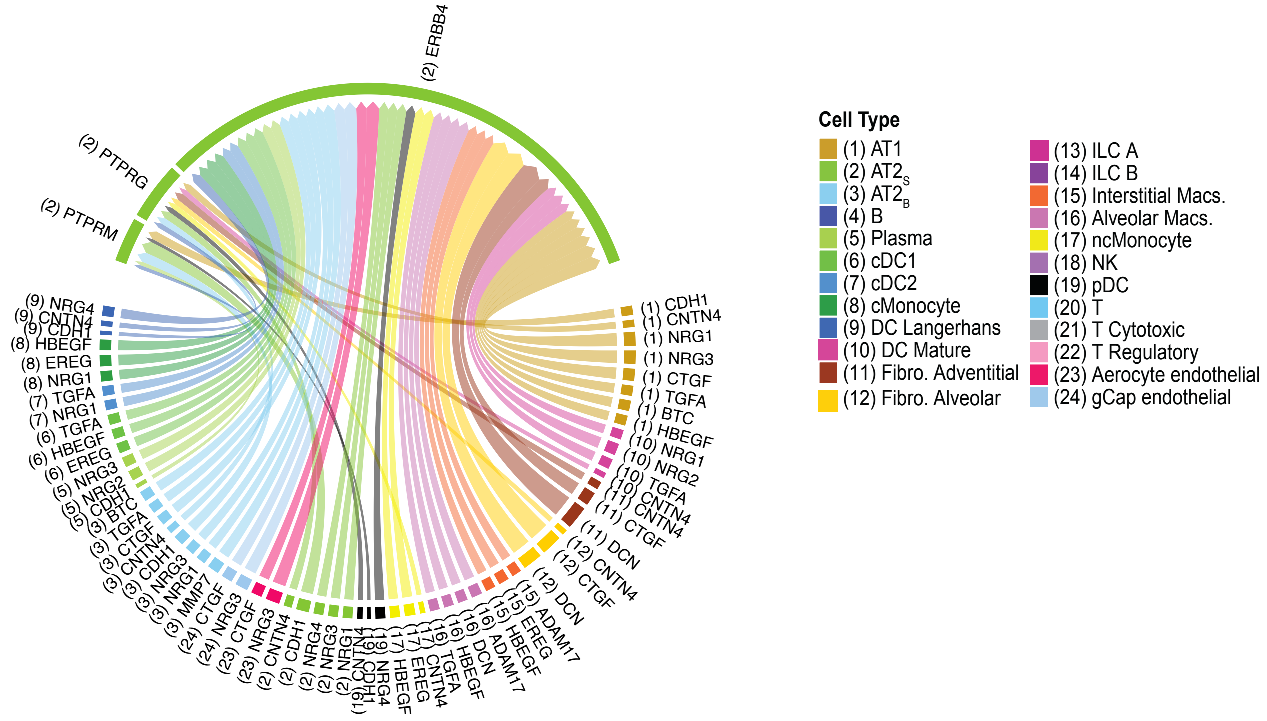

C

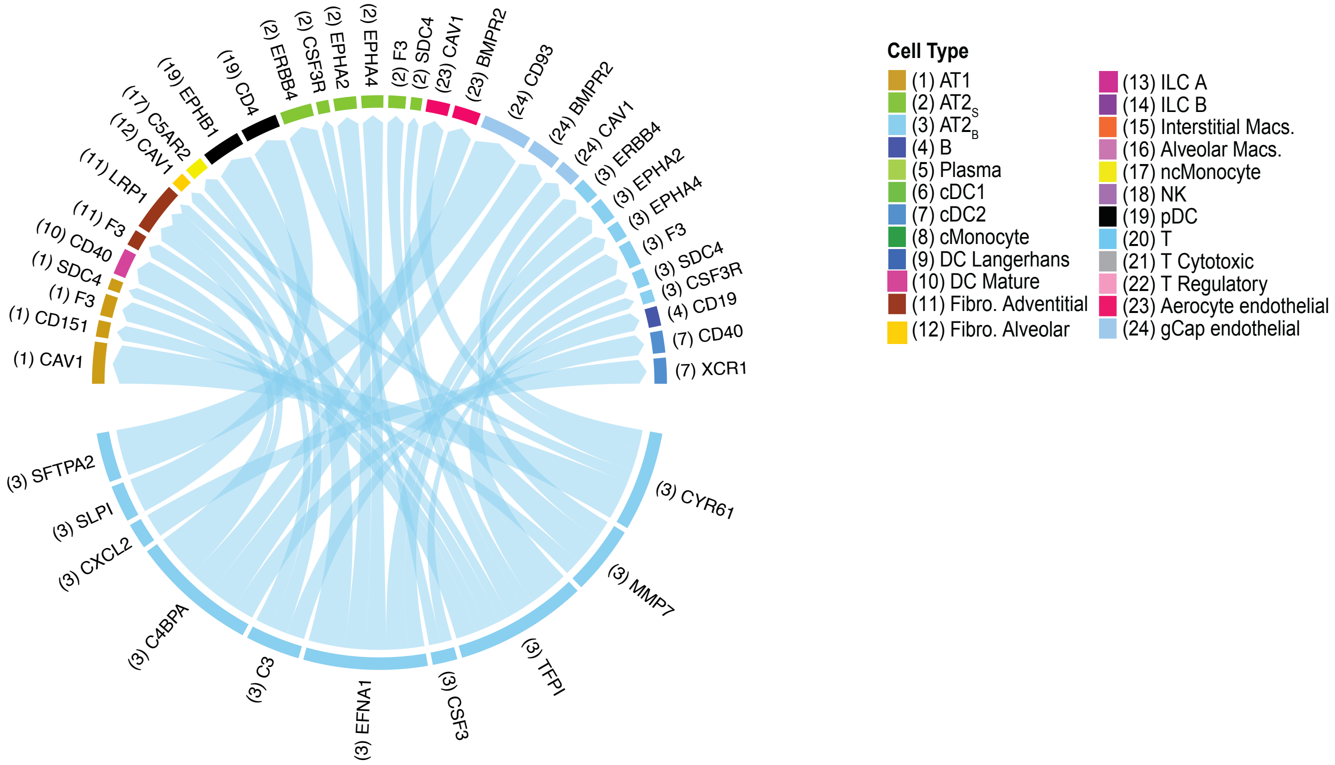

(19) EPHB1
(19) CD4
(2) ERBB4
(2) CSF3R
(2) EPHA2
(2) EPHA4
(2) F3
(2) SDC4
(23) CAV1
(23) BMPR2
(24) CD93
(24) BMPR2
(24) CAV1
(3) ERBB4
(3) EPHA2
(3) EPHA4
(3) F3
(3) SDC4
(3) CSF3R
(4) CD19
(7) CD40
(7) XCR1
(17) C5AR2
(12) CAV1
(11) LRP1
(11) F3
(10) CD40
(1) SDC4
(1) F3
(1) CD151
(1) CAV1
(3) SFTPA2
(3) SLPI
(3) CXCL2
(3) C4BPA
(3) C3
(3) EFNA1
(3) CSF3
(3) TFPI
(3) MMP7
(3) CYR61
Cell Type
(1) AT1
(2) AT2$_S$
(3) AT2$_B$
(4) B
(5) Plasma
(6) cDC1
(7) cDC2
(8) cMonocyte
(9) DC Langerhans
(10) DC Mature
(11) Fibro. Adventitial
(12) Fibro. Alveolar
(13) ILC A
(14) ILC B
(15) Interstitial Macs.
(16) Alveolar Macs.
(17) ncMonocyte
(18) NK
(19) pDC
(20) T
(21) T Cytotoxic
(22) T Regulatory
(23) Aerocyte endothelial
(24) gCap endothelial

D

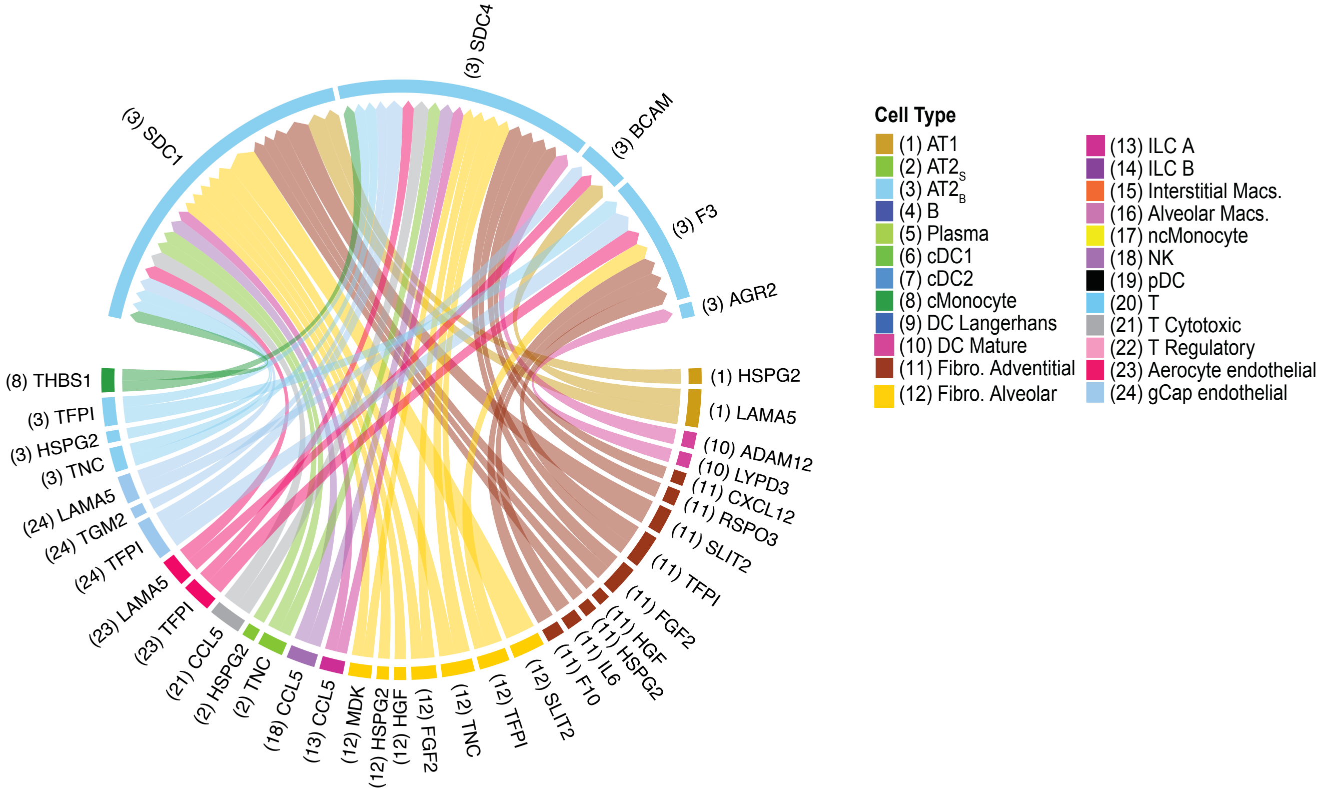

(3) SDC4
(3) SDC1
(3) BCAM
(3) F3
(3) AGR2
(8) THBS1
(3) TFPI
(3) HSPG2
(3) TNC
(24) LAMA5
(24) TGM2
(24) TFPI
(23) LAMA5
(23) TFPI
(21) CCL5
(2) HSPG2
(2) TNC
(18) CCL5
(13) CCL5
(12) MDK
(12) HSPG2
(12) HGF
(12) FGF2
(12) TNC
(12) TFPI
(12) SLIT2
(11) F10
(11) IL6
(11) HSPG2
(11) HGF
(11) FGF2
(11) TFPI
(11) SLIT2
(11) RSPO3
(11) CXCL12
(10) LYPD3
(10) ADAM12
(1) LAMA5
(1) HSPG2
Cell Type
(1) AT1
(2) AT2$_S$
(3) AT2$_B$
(4) B
(5) Plasma
(6) cDC1
(7) cDC2
(8) cMonocyte
(9) DC Langerhans
(10) DC Mature
(11) Fibro. Adventitial
(12) Fibro. Alveolar
(13) ILC A
(14) ILC B
(15) Interstitial Macs.
(16) Alveolar Macs.
(17) ncMonocyte
(18) NK
(19) pDC
(20) T
(21) T Cytotoxic
(22) T Regulatory
(23) Aerocyte endothelial
(24) gCap endothelial

Supplemental Figure 18: Pathway centrality analysis. Centrality analysis comparing the alveolar connectome for each mode of signalling between control (n=15) and COPD (n=17) with a minimal fold change > 0.3 for cell types with the top 3 Kleinberg centrality scores for each signaling mode. Panel shows outgoing edge weights and Kleinberg hub (outgoing) scores (*left*) and incoming edge weights and Kleinberg authority (incoming) scores (*right*). Cumulative edge weights (*x-axis*) have been scaled for each mode of signalling. Dot size is proportional to the Kleinberg scores for each cell type within a given mode of signalling. Individual cell types are color labelled and numbers shown identify cell types with the largest cumulative edge weights. **** P<0.0001, *** P<0.001, ** P<0.01, * P<0.05 using the two-sided Durbin test to compare control and COPD across cell types for each signalling mode. Interleuk = Interleukins, Semaph = Semaphorins. AT1 = alveolar epithelial type I cells, AT2 = alveolar epithelial type II cells, PNEC = pulmonary neuroendocrine cells, SMC = smooth muscle cells, gCap = general capillary, cMonocyte = classical monocytes, ncMonocyte = non-classical monocyte, Macs. = macrophages, DC = dendritic cells, cDC = conventional dendritic cells, pDC = plasmacytoid dendritic cells, NK = natural killer cells, ILC = innate lymphoid cells.

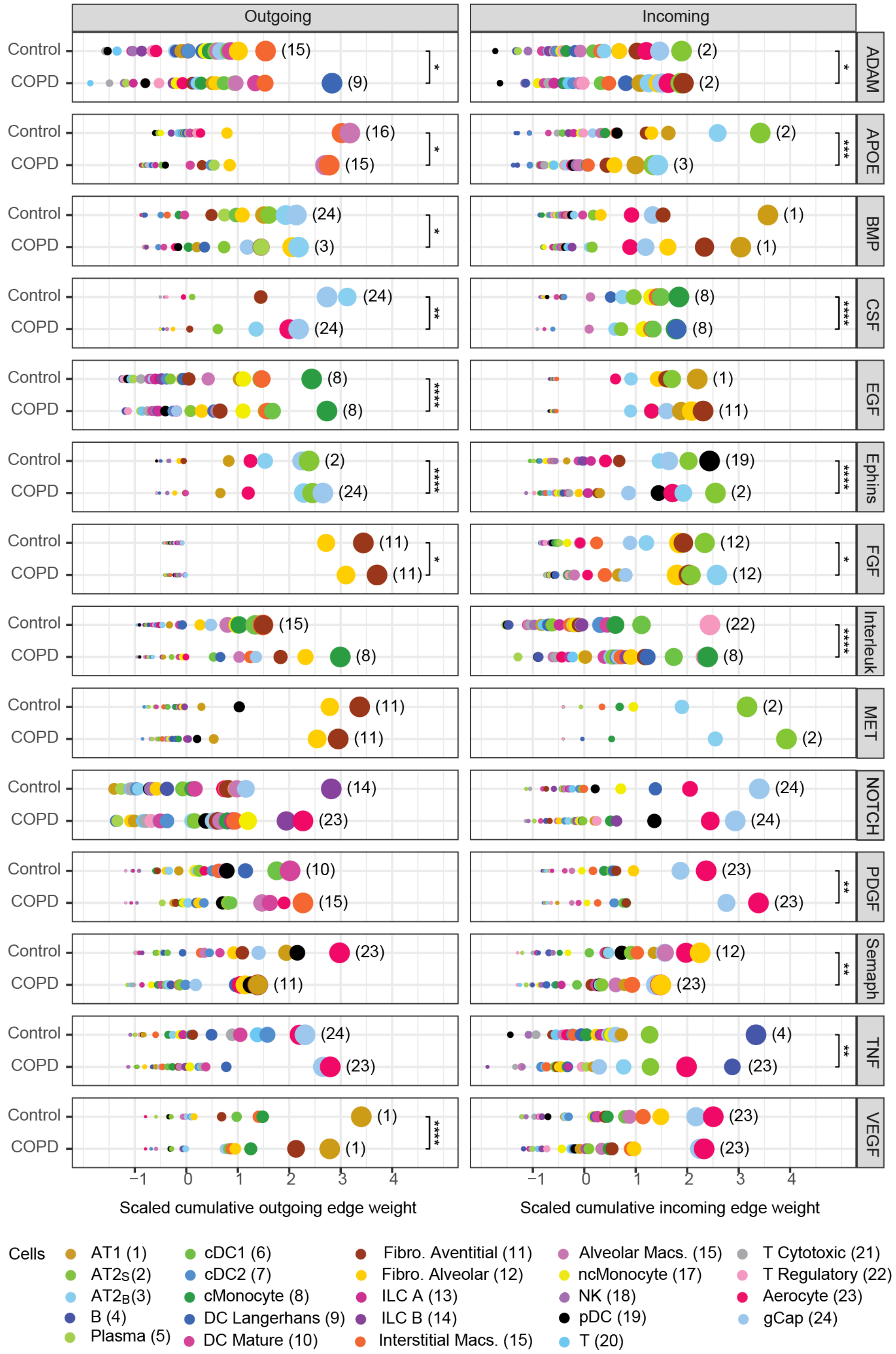

Supplemental Figure 19: CXCL connectome in human subjects. CXCL connectome comparing never smokers without COPD (n=11), current/former smokers without COPD (n=4), and subjects with COPD (n=17) demonstrating an increase in outgoing CXCL signaling from gCap endothelial cells. Dot size is proportional to the Kleinberg centrality scores for each cell type within a given mode of signalling. Individual cell types are color labelled and numbers shown identify cell types with the largest cumulative edge-weights. **** P<0.001 using the two-sided Durbin test to compare control and COPD across cell types for each signalling mode. Notably, there were no pDC cells detected amongst current/former smokers without COPD and therefore pDCs have been excluded from this analysis. AT1 = alveolar epithelial type I cells, AT2 = alveolar epithelial type II cells, PNEC = pulmonary neuroendocrine cells, SMC = smooth muscle cells, gCap = general capillary, cMonocyte = classical monocytes, ncMonocyte = non-classical monocyte, Macs. = macrophages, DC = dendritic cells, cDC = conventional dendritic cells, pDC = plasmacytoid dendritic cells, NK = natural killer cells, ILC = innate lymphoid cells.

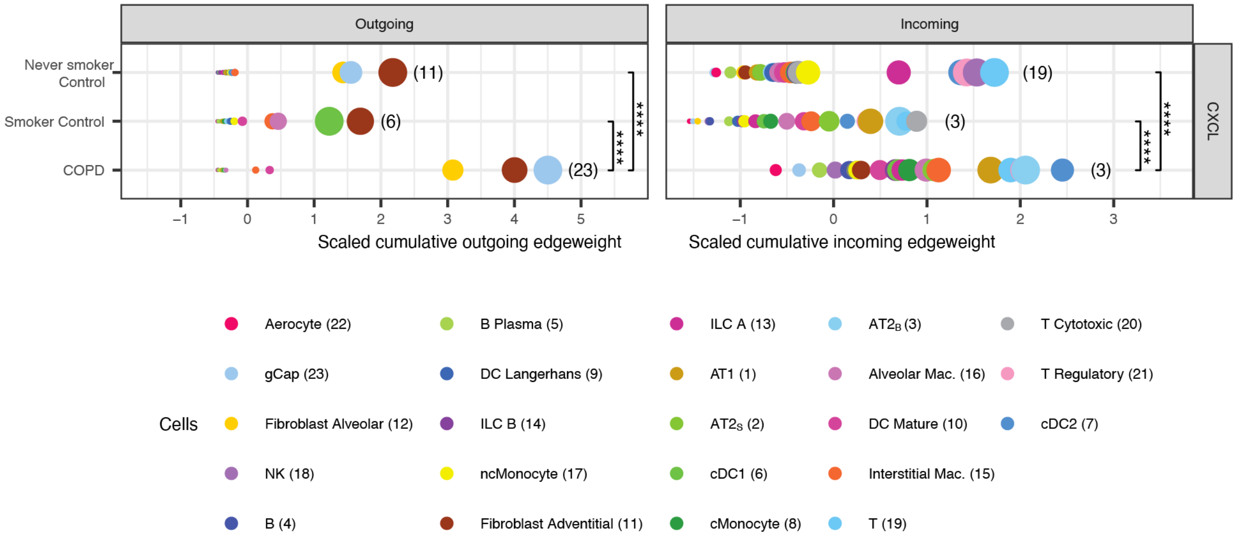

!

Supplemental Figure 20: CXCL connectome in mice. CXCL connectome for 2 male and 2 female mice exposed to room air (RA) or 2 male and 2 female cigarette smoke (CS) demonstrating that the largest relative increase in outgoing CXCL signaling is from gCap endothelial cells. Dot size is proportional to the Kleinberg centrality scores for each cell type within a given mode of signalling. Individual cell types are colour labelled and numbers shown identify cell types with the largest cumulative edge-weights. *** P<0.001 using the two-sided Durbin test to compare control and COPD across cell types for each signalling mode. AT1 = alveolar epithelial type I cells, AT2 = alveolar epithelial type II cells, PNEC = pulmonary neuroendocrine cells, SMC = smooth muscle cells, gCap = general capillary, cMonocyte = classical monocytes, ncMonocyte = non-classical monocyte, Macs. = macrophages, DC = dendritic cells, cDC = conventional dendritic cells, pDC = plasmacytoid dendritic cells, NK = natural killer cells, ILC = innate lymphoid cells, Fibro = fibroblast.

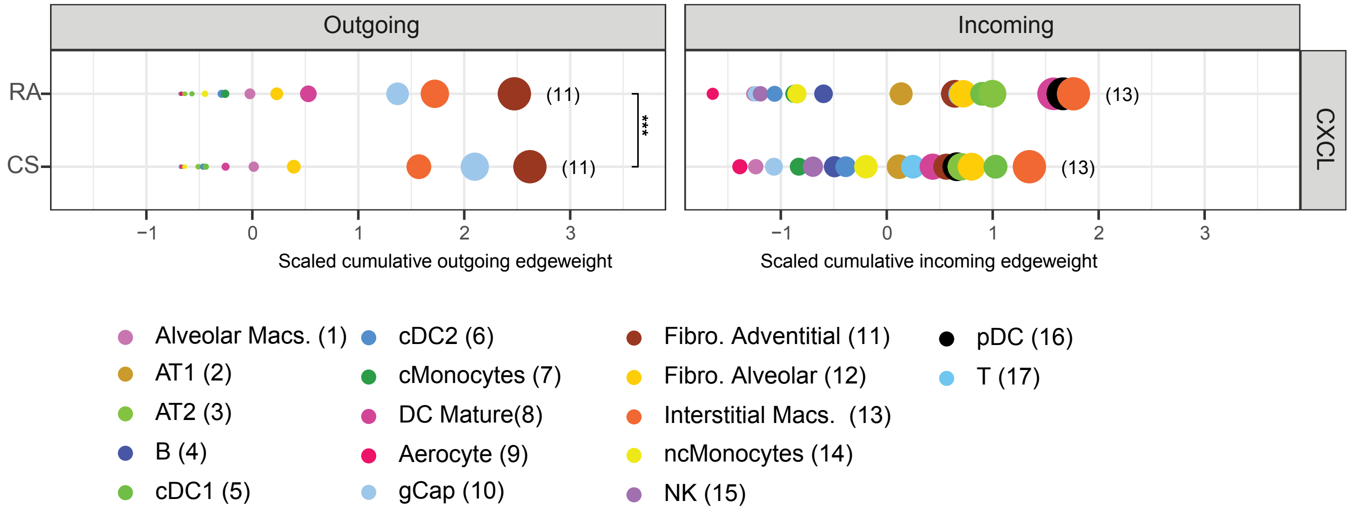

.

Supplemental Figure 21: CXCL12 signaling. Differential circos plots of CXCL signaling demonstrating increases in outgoing CXCL signaling comparing **A)** current/former smokers without COPD vs. never smokers without COPD, **B**) COPD subjects vs. never smokers without COPD, and **C**) COPD subjects vs. current/former smokers w/o COPD. AT1 = alveolar epithelial type I cells, AT2 = alveolar epithelial type II cells, PNEC = pulmonary neuroendocrine cells, SMC = smooth muscle cells, gCap = general capillary, cMonocyte = classical monocytes, ncMonocyte = non-classical monocyte, Macs. = macrophages, DC = dendritic cells, cDC = conventional dendritic cells, pDC = plasmacytoid dendritic cells, NK = natural killer cells, ILC = innate lymphoid cells.

A

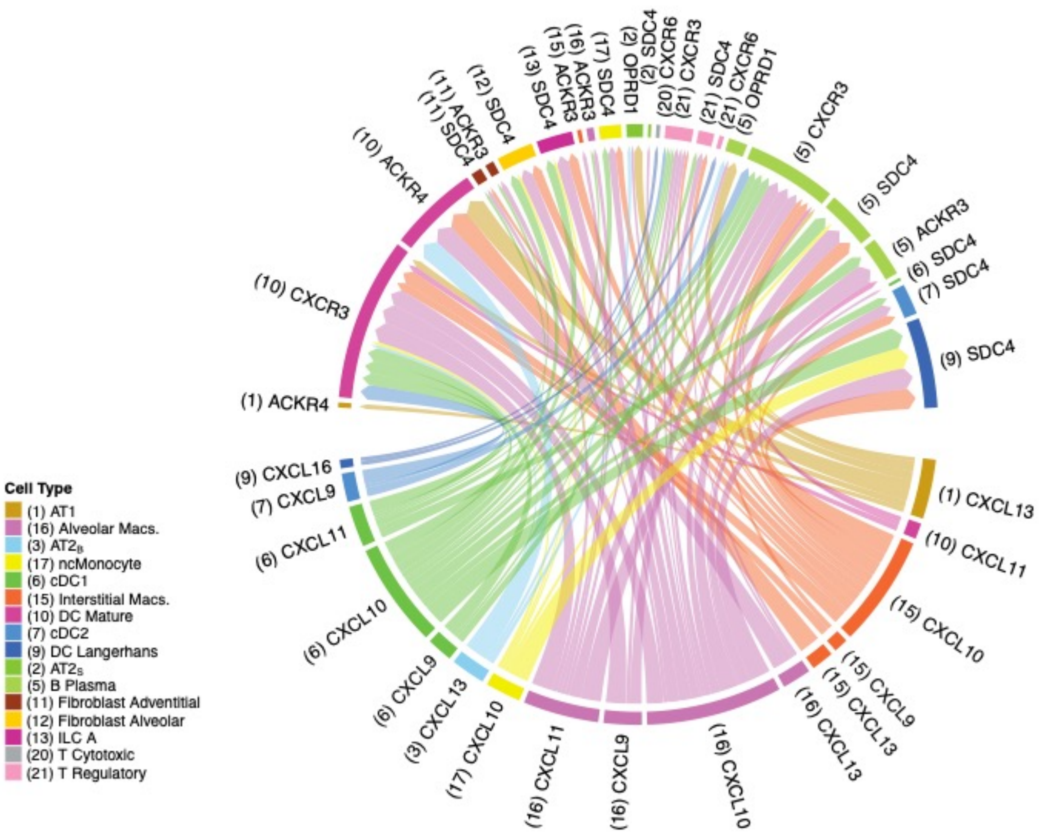

B

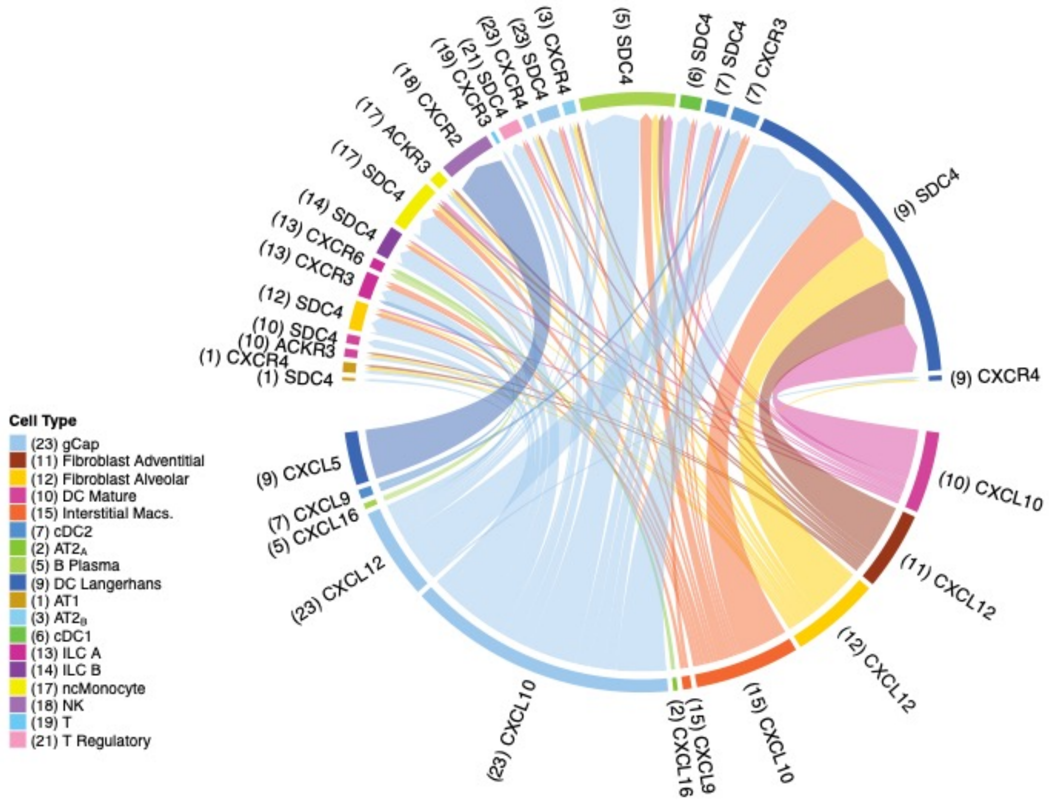
Cell Type
(23) gCap
(11) Fibroblast Adventitial
(12) Fibroblast Alveolar
(10) DC Mature
(15) Interstitial Macs.
(7) cDC2
(2) AT2A
(5) B Plasma
(9) DC Langerhans
(1) AT1
(3) AT2B
(6) cDC1
(13) ILC A
(14) ILC B
(17) ncMonocyte
(18) NK
(19) T
(21) T Regulatory
(9) SDC4
(9) CXCR4
(10) CXCL10
(11) CXCL12
(12) CXCL12
(15) CXCL10
(15) CXCL9
(2) CXCL16
(23) CXCL10
(23) CXCL12
(5) CXCL16
(7) CXCL9
(9) CXCL5
(1) SDC4
(1) CXCR4
(10) ACKR3
(10) SDC4
(12) SDC4
(13) CXCR3
(13) CXCR6
(14) SDC4
(17) SDC4
(17) ACKR3
(18) CXCR2
(19) CXCR3
(21) SDC4
(23) CXCR4
(23) SDC4
(3) CXCR4
(5) SDC4
(6) SDC4
(7) SDC4
(7) CXCR3

C

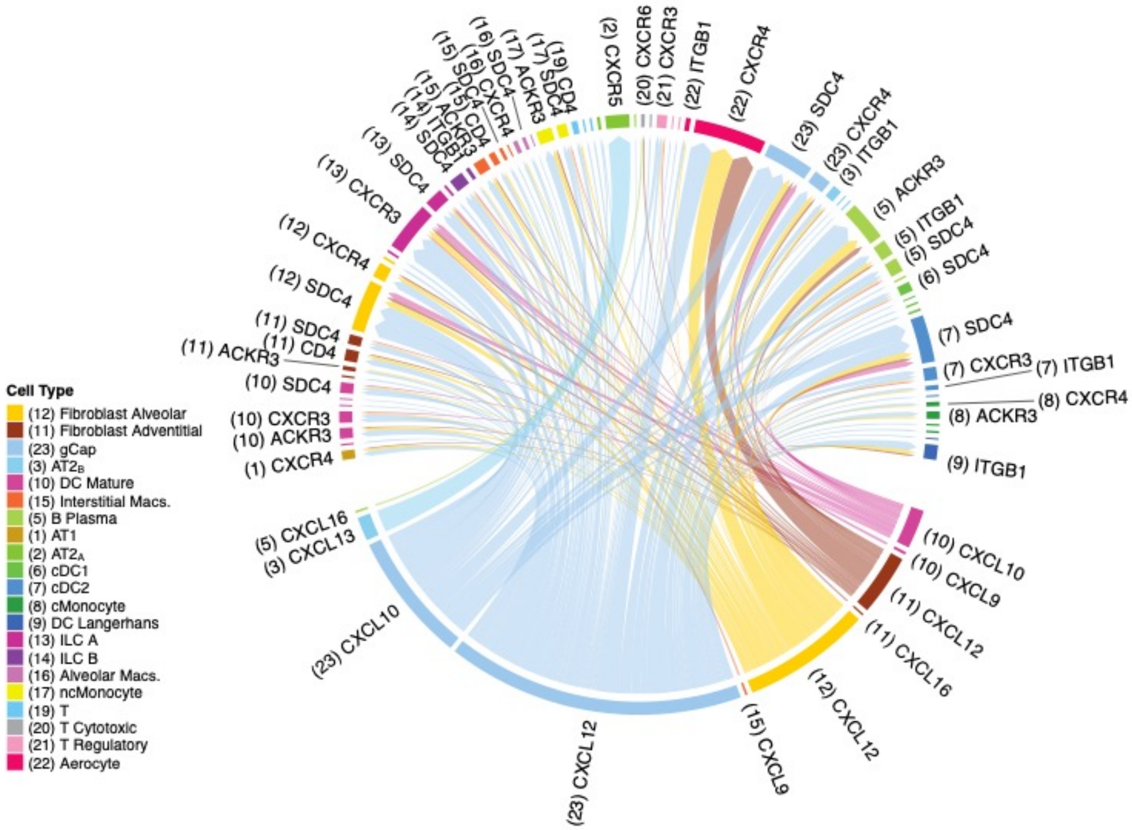
Cell Type
(12) Fibroblast Alveolar
(11) Fibroblast Adventitial
(23) gCap
(3) AT2B
(10) DC Mature
(15) Interstitial Macs.
(5) B Plasma
(1) AT1
(2) AT2A
(6) cDC1
(7) cDC2
(8) cMonocyte
(9) DC Langerhans
(13) ILC A
(14) ILC B
(16) Alveolar Macs.
(17) ncMonocyte
(19) T
(20) T Cytotoxic
(21) T Regulatory
(22) Aerocyte
(2) CXCR5
(20) CXCR6
(21) CXCR3
(22) ITGB1
(22) CXCR4
(23) SDC4
(23) CXCR4
(3) ITGB1
(5) ACKR3
(5) ITGB1
(5) SDC4
(6) SDC4
(7) SDC4
(7) CXCR3
(7) ITGB1
(8) CXCR4
(8) ACKR3
(9) ITGB1
(10) CXCL10
(10) CXCL9
(11) CXCL12
(11) CXCL16
(12) CXCL12
(15) CXCL9
(23) CXCL12
(23) CXCL10
(3) CXCL13
(5) CXCL16
(1) CXCR4
(10) ACKR3
(10) CXCR3
(10) SDC4
(11) ACKR3
(11) CD4
(11) SDC4
(12) SDC4
(12) CXCR4
(13) CXCR3
(13) SDC4
(19) CD4
(17) SDC4
(17) ACKR3
(16) CXCR4
(16) SDC4

Supplemental Figure 22: *CXCL12* in capillary endothelial cells. Immunofluorescence staining of NOSTRIN (aqua) and PRX (green), *in situ* hybridization for *CXCL12* mRNA (red), and DAPI (blue) in control and COPD lung tissue. Bar = 100 μm. Original magnification ×20. Images representative of 5 control and 5 COPD samples.

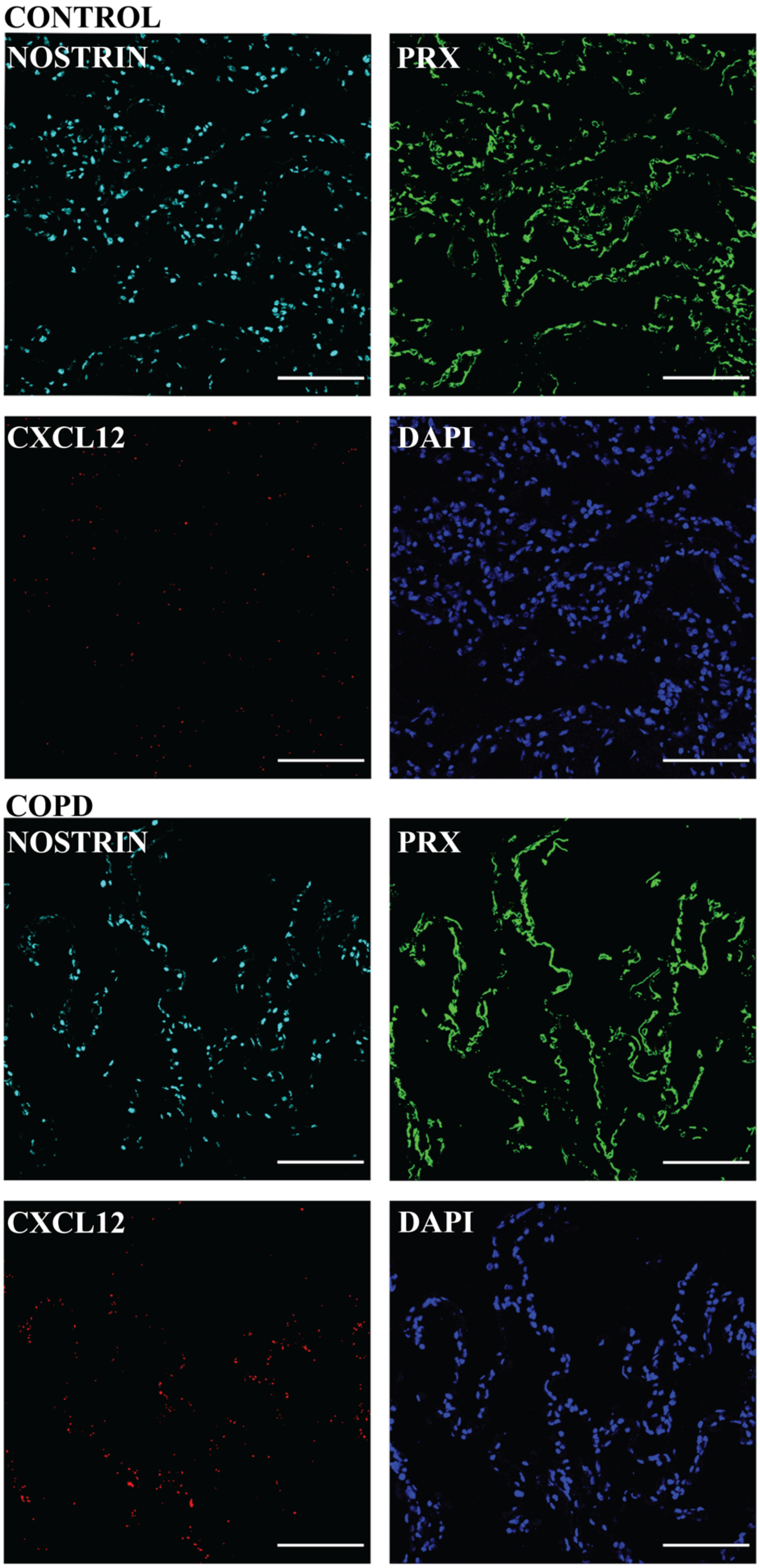

Supplemental Figure 23: Immune cell populations. A) UMAPs of cell types (*left*) and disease phenotype (*right*) for immune cells in our human scRNAseq dataset. **B,C)** Boxplots of cellular proportions present in COPD and control lung tissues based on fraction of the number of cells per subject for each broad cell type grouping present within the single cell dataset. **B,C)** Boxplots of cellular proportions present in COPD and control lung tissues based on fraction of the number of cells per subject for **B)** myeloid cells and **C)** lymphoid cells within the single cell dataset (n = 17 COPD subjects and 15 control subjects). Boxes represent median and interquartile ranges (IQRs); whiskers are 1.5 x IQR and dots represent subjects outside the IQR range. cMonocyte = classical Mϕ monocytes, ncMonocyte = non-classical monocyte, Mϕ = interstitial macrophages, Mϕ alveolar = alveolar macrophages, DC = dendritic cells, cDC = conventional dendritic cells, pDC = plasmacytoid dendritic cells, NK = natural killer cells, ILC = innate lymphoid cells.

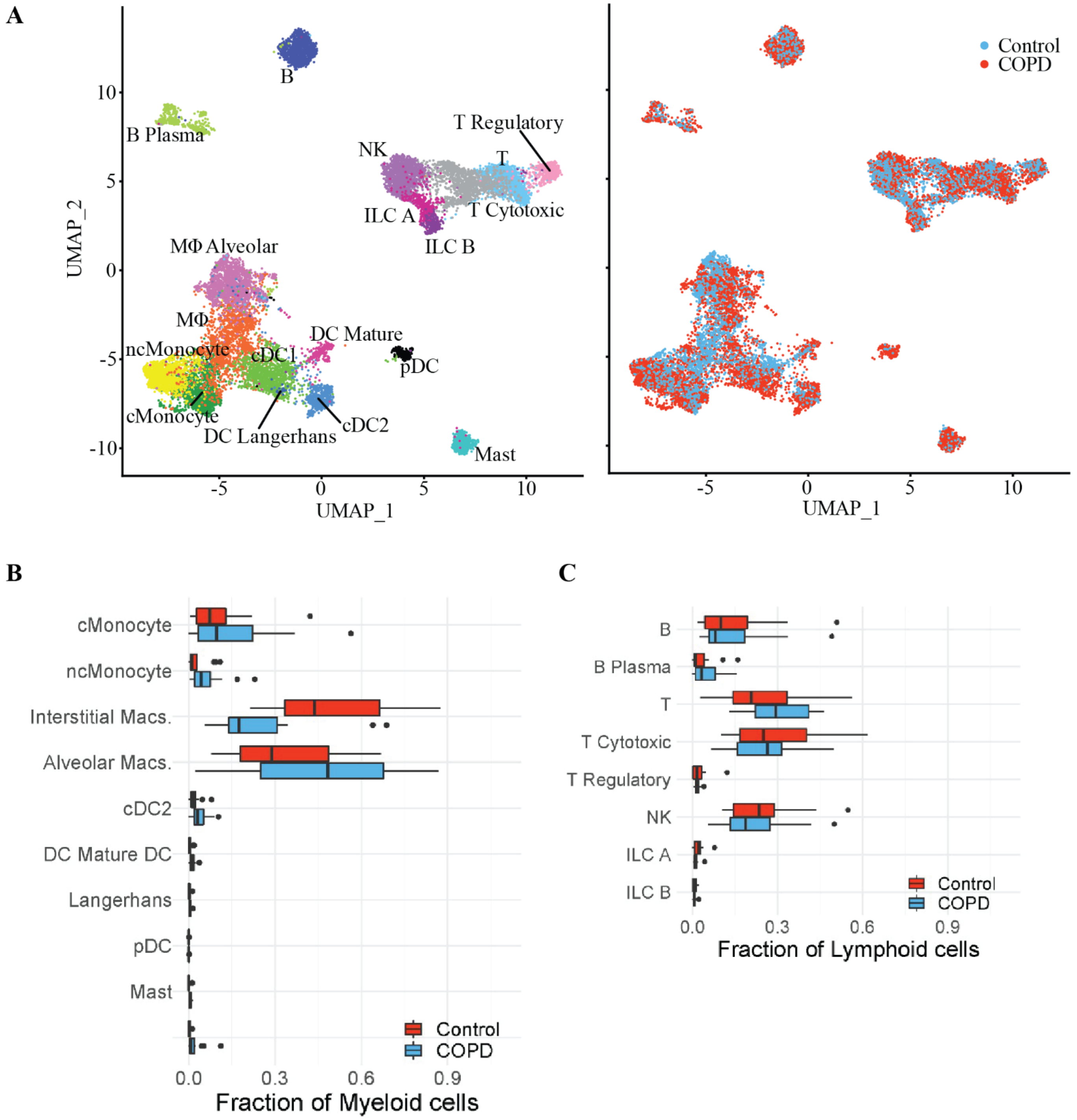

Supplemental Figure 24: **Percent makeup of alveolar macrophages by cluster.** Percent makeup of alveolar macrophage across all nine Louvain alveolar macrophage clusters per subject, from never smokers without COPD (n=10), current/former smokers without COPD (n=3), and subjects with COPD (n=14). Bar plots represent median, whiskers represent interquartile ranges (IQR). All dots, representing subjects, are shown.

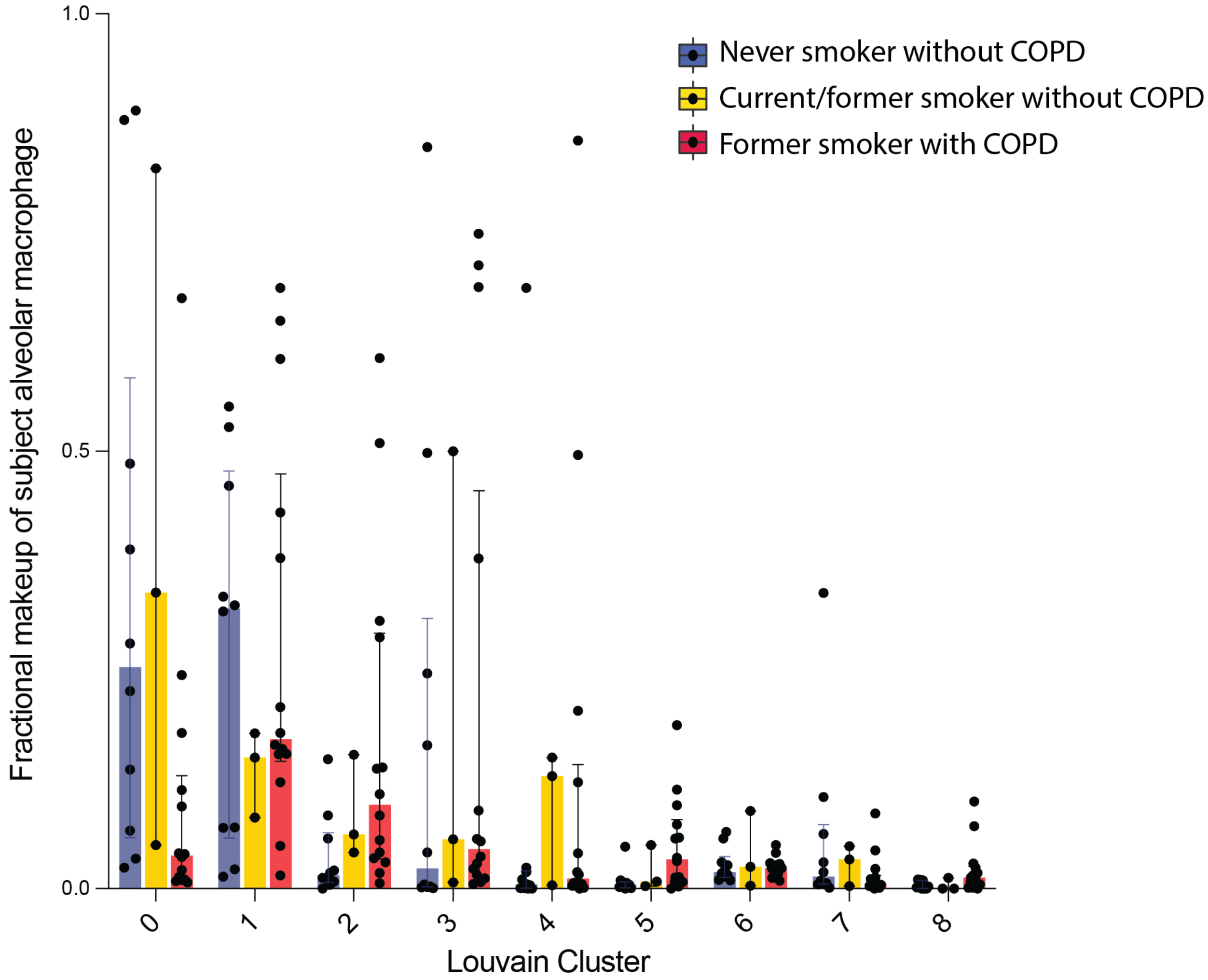

Supplemental Figure 25: MT2A expression in CD68+ macrophages. Immunofluorescence staining of CD68$^+$ macrophages(green), MT2A (red), and, DAPI (blue) in control and COPD lung tissue. Bar = 100 μm. Original magnification ×20**.** Images representative of 5 control and 5 COPD samples.

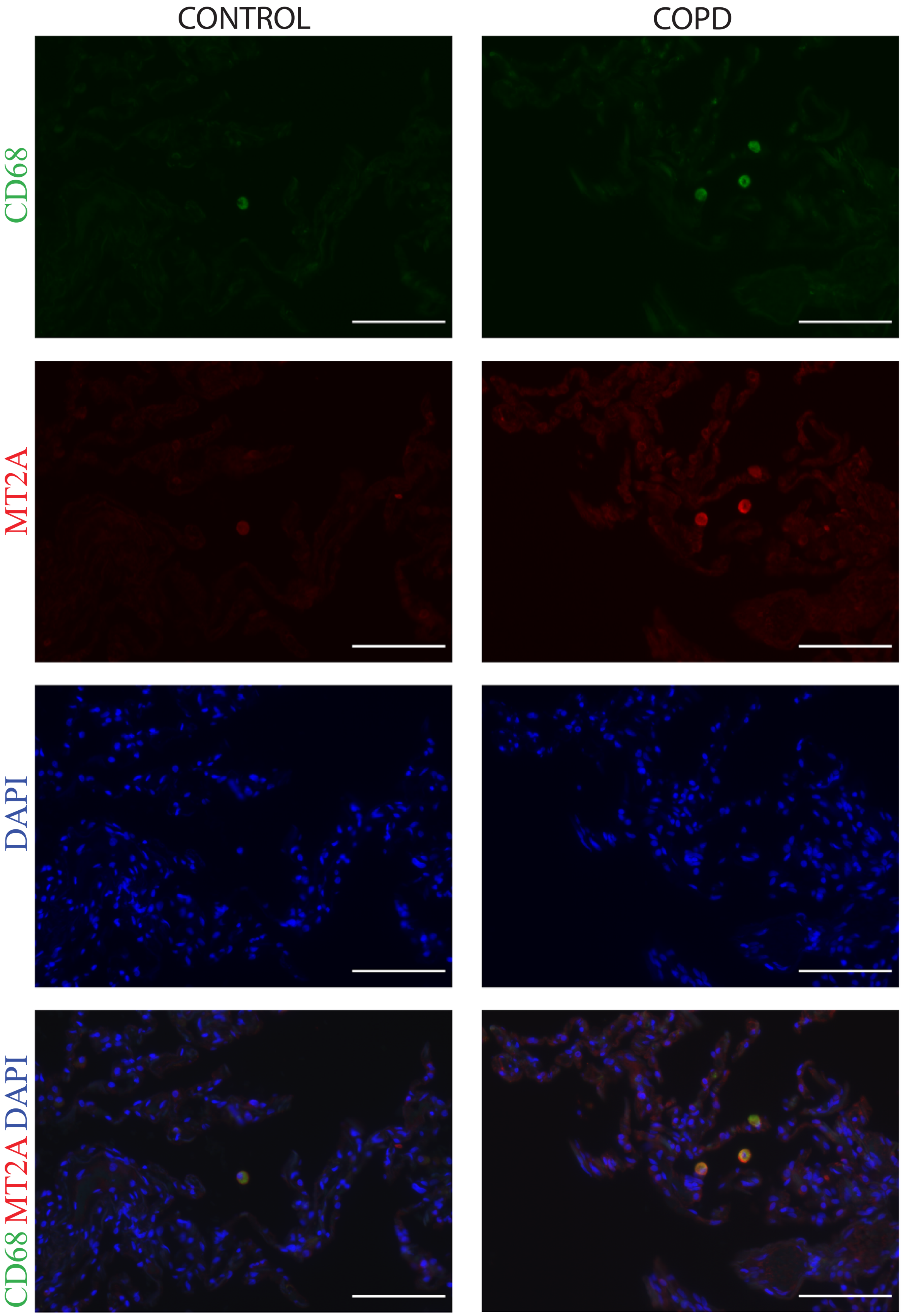

Supplemental Figure 26: Alveolar macrophage expression of *THBS1, PELI1*, and *CDC42*.
Density plots of *THBS1, PELI1,* and *CDC42* normalized gene expression across all alveolar macrophage cells, grouped by control (blue) and COPD (red) subjects. Within each disease group, subjects are ordered top to bottom from highest within-disease group expression average to lowest.

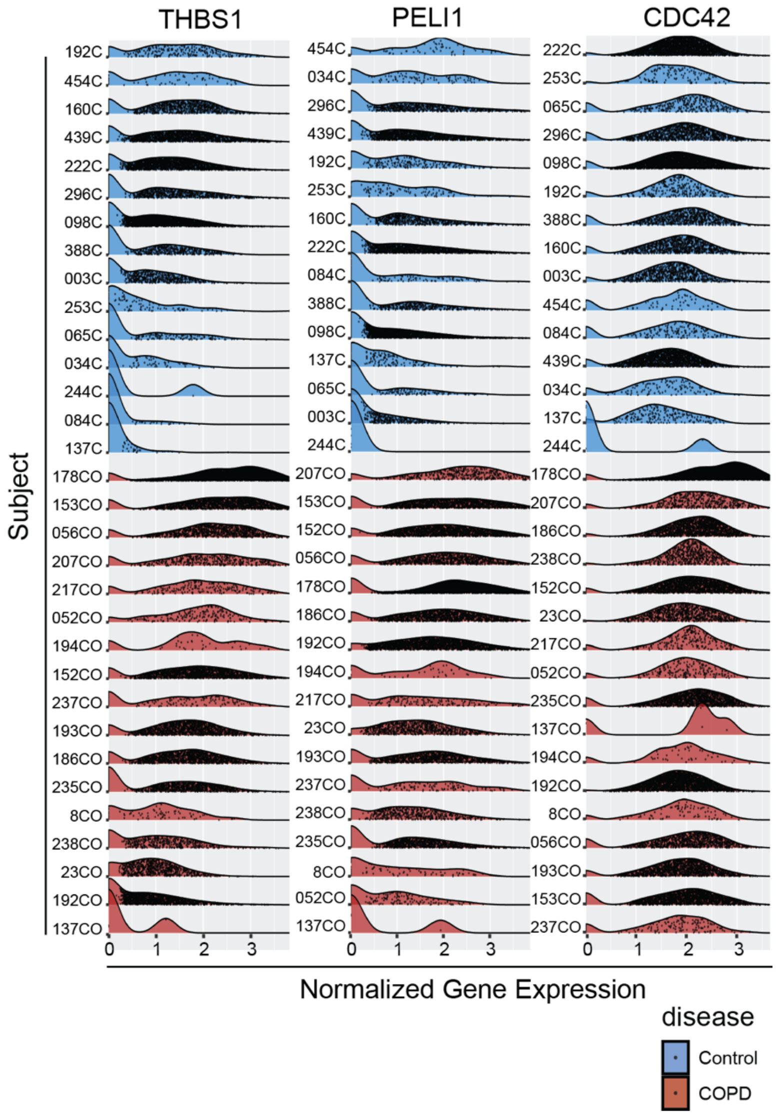

Supplement: Supplementary file 1 — Supplementary Information [file 41467_2022_28062_MOESM1_ESM.pdf]
