# Supplementary material for: Characterization of the COPD alveolar niche using single-cell RNA sequencing
Source: Nat Commun. 2022 Jan 25;13:494. doi: 10.1038/s41467-022-28062-9 (PMC8789871; doi:10.1038/s41467-022-28062-9)
Supplement: Supplementary file 3 — Description of Additional Supplementary Files [file 41467_2022_28062_MOESM3_ESM.docx]

Title: Supplementary Dataset 1

Description: This file contains the output of the CELLEX analysis, which represents the specificity likelihood for each gene across different cell types

Title: Supplementary Dataset 2

Description: Pathway specific changes in cell-cell signaling in human and mouse datasets with COPD (human) and 10 months of cigarette smoke exposure (mouse), respectively.
