# Supplementary material for: Characterization of the COPD alveolar niche using single-cell RNA sequencing
Source: Nat Commun. 2022 Jan 25;13:494. doi: 10.1038/s41467-022-28062-9 (PMC8789871; doi:10.1038/s41467-022-28062-9)
Supplement: Supplementary file 6 — Reporting Summary [file 41467_2022_28062_MOESM6_ESM.pdf]

nature research

Corresponding author(s): Maor Sauler
John McDonough

Last updated by author(s): November 17, 2021

Reporting Summary

Nature Research wishes to improve the reproducibility of the work that we publish. This form provides structure for consistency and transparency in reporting. For further information on Nature Research policies, see our Editorial Policies and the Editorial Policy Checklist.

Statistics

For all statistical analyses, confirm that the following items are present in the figure legend, table legend, main text, or Methods section.

n/a	Confirmed	
☐	☒	The exact sample size (*n*) for each experimental group/condition, given as a discrete number and unit of measurement
☐	☒	A statement on whether measurements were taken from distinct samples or whether the same sample was measured repeatedly
☐	☒	The statistical test(s) used AND whether they are one- or two-sided<br>*Only common tests should be described solely by name; describe more complex techniques in the Methods section.*
☐	☒	A description of all covariates tested
☐	☒	A description of any assumptions or corrections, such as tests of normality and adjustment for multiple comparisons
☐	☒	A full description of the statistical parameters including central tendency (e.g. means) or other basic estimates (e.g. regression coefficient) AND variation (e.g. standard deviation) or associated estimates of uncertainty (e.g. confidence intervals)
☐	☒	For null hypothesis testing, the test statistic (e.g. *F*, *t*, *r*) with confidence intervals, effect sizes, degrees of freedom and *P* value noted<br>*Give P values as exact values whenever suitable.*
☒	☐	For Bayesian analysis, information on the choice of priors and Markov chain Monte Carlo settings
☒	☐	For hierarchical and complex designs, identification of the appropriate level for tests and full reporting of outcomes
☐	☒	Estimates of effect sizes (e.g. Cohen's *d*, Pearson's *r*), indicating how they were calculated

Our web collection on statistics for biologists contains articles on many of the points above.

Software and code

Policy information about availability of computer code

Data collection	mkfastq pipeline in Cell Ranger's (v3.0.2). cutadapt (v1.17 and v2.9), zUMIs pipeline (v2.0), TrimGalore! (v0.6.6), STAR (v2.6.0c, v2.7.3a, and v2.7.5c)
Data analysis	Single-cell analysis was performed using the Seurat R package (v3.2.3 and v4.0.4) using the recommended workflow.<br>CELLEX (v.1.2.1) CELLEX: (https:// github.com/perslab/CELLEX) was performed using recommended normalization method and preprocessing steps. We then ran CELLECT v. 1.1.0 with the recommended workflow (CELLECT-LDSC) and default parameters (100 kb window size around each gene) (https://github.com/perslab/ CELLECT). Connectome analysis was performed using R software Connectome (vl.0.0) https://msraredon.github.io/Connectome/). Average expression values for every gene within cell types were calculated and mapped against the FANTOMS database of known ligand-receptor pairs to create a global connectome. For the centrality figure, the code we used was very similar to the CompareCentrality function in the Connectome (v l.0.0) package, but we changed the scaling method for visualization and added the Durbin significance test feature. The function for the network maps is not included in the Connectome package, so we added it. Finally, for the fold change calculations, the method was previously described , but the code was separate from the Connectome package. Modifications available to readers upon request.<br>Other: Fiji v.1.0, GraphPad Prism v9.3.0, CellProfiler v4.2.1, featureCounts (v2.0.1), UpsetR(v.1.4)

For manuscripts utilizing custom algorithms or software that are central to the research but not yet described in published literature, software must be made available to editors and reviewers. We strongly encourage code deposition in a community repository (e.g. GitHub). See the Nature Research guidelines for submitting code & software for further information.

Data

Policy information about availability of data

All manuscripts must include a data availability statement. This statement should provide the following information, where applicable:

- Accession codes, unique identifiers, or web links for publicly available datasets
- A list of figures that have associated raw data
- A description of any restrictions on data availability

Data Availability: The datasets generated during and/or analyzed during the current study are available or have been deposited on NCBI Gene Expression Omnibus. Specifically, Human scRNAseq data is available under accession code GSE136831 (https://www.ncbi.nlm.nih.gov/geo/query/acc.cgi?acc=GSE136831). Mouse scRNAseq is available under accession code GSE168299 (https://www.ncbi.nlm.nih.gov/geo/query/acc.cgi?acc=GSE168299) . LGRC data is available is available under accession code GSE47460 (https://www.ncbi.nlm.nih.gov/geo/query/acc.cgi?acc=GSE47460). Human Single-cell expression data can also be interactively explored online at www.COPDcellatlas.com. A complete list of genome-wide association summary statistics are available at the database of Genotypes and Phenotypes (dbGaP) under accession phs000179.v6.p2 for COPD (defined as FEV1/FVC ratio) and UK Biobank GWAS summary statistics for FEV1/FVC ratio are available at www.ebi.ac.uk/gwas, study accession GCST007431. Mouse reference genome GRCm38 release M22 (GRCm38.p6) was downloaded from GENCODE (www.genecodegenes.org) and the human genome reference GRCh38 release 91 was downloaded from ENSEMBLE www.ensemble.org. Source data are provided with this paper.

Field-specific reporting

For a reference copy of the document with all sections, see nature.com/documents/nr-reporting-summary-flat.pdf

Please select the one below that is the best fit for your research. If you are not sure, read the appropriate sections before making your selection.

☒ Life sciences ☐ Behavioural & social sciences ☐ Ecological, evolutionary & environmental sciences

Life sciences study design

All studies must disclose on these points even when the disclosure is negative.

Sample size	No sample size calculation was performed for human studies because we used available samples for analysis and sample size is restricted due to practical constraints related to availability of explanted lung tissue. We chose to use both male and female mice for murine studies but limited samples size to 2 mice/gender/group due to cost of single-cell RNA seq analysis and limited resources.
Data exclusions	All data exclusion occurred prior to data analysis. For human scRNAseq, one COPD sample was excluded due to a reported history of no cigarette smoke exposure. In order to age-match our control and COPD samples, we excluded control samples from individuals < 40 years of age. For human single-cell RNA sequencing , we removed cells with 12% of transcripts arising from unspliced RNA, cells with less than 1000 transcripts profiled or > 20% of their transcriptome of mitochondrial origin were then removed. For mice: We removed barcoded cells with <7.5% of transcripts arising from unspliced mRNA, cells with <1000 transcripts profiled, and cells with >5% of their transcriptome of mitochondrial origin. Background contamination from cell free mRNA was removed using SoupX software (v1.2.2). Finally, in Figure 6B and supplemental Figure 24, we excluded samples where the number of alveolar macrophages < 100.
Replication	The number of biological replicates in each experiment is described in the Methods section and figure legends. Key findings from paper were reproduced, either in other human cohorts and/or in murine studies. Key findings were also validated with immunofluorescence or in situ hybridization staining.
Randomization	Mice were randomized to either receive cigarette smoke exposure or room air exposure. Human samples were not randomized due to patient recruitment and sample collection based on availability of donor lung tissue. For experiments other than those on mice and human samples, samples were randomly allocated into experimental groups.
Blinding	For human samples, blinding was not appropriate as groups were assigned based on disease. Similar, the investigator could not be blinded as to whether the mice received CS exposure or not. However, computational analyses, such as clustering did not use experimental group to assign annotation. For immunofluorescence staining and in situ hybridization experiments, microscopists were blinded to whether the samples were from control or COPD patients. For other in vitro experiments, investigators could not be blinded to their own experimental groups.

Reporting for specific materials, systems and methods

We require information from authors about some types of materials, experimental systems and methods used in many studies. Here, indicate whether each material, system or method listed is relevant to your study. If you are not sure if a list item applies to your research, read the appropriate section before selecting a response.

Materials & experimental systems

n/a	Involved in the study	
☐	☒	Antibodies
☐	☒	Eukaryotic cell lines
☒	☐	Palaeontology and archaeology
☐	☒	Animals and other organisms
☐	☒	Human research participants
☒	☐	Clinical data
☒	☐	Dual use research of concern

Methods

n/a	Involved in the study	
☒	☐	ChIP-seq
☐	☒	Flow cytometry
☒	☐	MRI-based neuroimaging

Antibodies

Antibodies used

1)mouse anti-MT2A (MAB10176) (R&D systems) (clone 1006811)(Lot CMGQ0119031) https://www.rndsystems.com/products/human-mt2a-antibody-1006811_mab10176
2)rabbit anti-CD68 (PA5-83940) (Invitrogen) (lot: vi23083913b)https://www.thermofisher.com/antibody/product/CD68-Antibody-Polyclonal/PA5-83940
3) rabbit anti-SFTPC, AB3786, Millipore (3537117)https://www.emdmillipore.com/US/en/product/Anti-Prosurfactant-Protein-C-proSP-C-Antibody,MM_NF-AB3786
4) mouse anti-SFTPC, sc-518029, Santa Cruz lot#C0519 https://www.scbt.com/p/sp-c-antibody-h-8
5) rabbit anti-NUPR1, ab#234696, Abcam, lot#GR3227521-18 https://www.abcam.com/p8nupr1-antibody-ab234696.html
6) rabbit anti-PRX, NBP1-89598, Novus (lot number 000008772) https://www.novusbio.com/products/periaxin-antibody_nbp1-89598
7)mouse anti-NOSTRIN, sc373954, Santa Cruz (Lot:I1517) https://www.scbt.com/p/nostrin-antibody-b-9

PE anti-human CD326 (EpCAM) (eBioscience), (Catalog number: 5011259, clone 1B7) https://www.thermofisher.com/antibody/product/CD326-EpCAM-Antibody-clone-1B7-Monoclonal/12-9326-42
FITC anti-human CD45 (BD Bioscience), (Catalog Number: 340664): Clone 2D1 (ASR) https://www.bdbiosciences.com/en-us/products/reagents/flow-cytometry-reagents/clinicl-diagnostics/single-color-antibodies-asr-ivd-ce-ivd/cd45-fitc.340664
Alexa Fluor® 647 anti-human podoplanin (PDPN) (clone LpMab-21) (BioLegend) (Catalog number: 395003): https://www.biolegend.com/en-us/products/alexa-fluor-647-anti-human-podoplanin-antibody-17095?GroupID=GROUP28

Validation

Antibody-specific validations are available as indicated on the manufacturers' web page. Links are listed in the "Antibodies Used" section above.

Eukaryotic cell lines

Policy information about cell lines

Cell line source(s)

A549 (#CCL-185) and SAECs (#PCS-301-010) were purchased from American Type Tissue Collection. iPSC-derived AT2 cells were obtained as described in the manuscript.

Authentication

Cell lines were not authenticated.

Mycoplasma contamination

Cell lines were not tested for mycoplasma contamination

Commonly misidentified lines
(See ICLAC register)

No commonly misidentified lines were used.

Animals and other organisms

Policy information about studies involving animals; ARRIVE guidelines recommended for reporting animal research

Laboratory animals

We obtained Sftpc-CreERT2 (stock #028054) and Rosa26-mTmG C57Bl/6 (stock #007676) mice from Jackson Laboratories and bred them together to generate Sftpc-Cre/Rosa26-mTmG mice. 2 Male and 2 female 8-10-week-old mice. Mouse were maintained in local housing facility of a controlled condition (23 ± 1 °C, 50 ± 10% humidity and 12–12 h light-dark cycle).

Wild animals

This study did not involve wild animals

Field-collected samples

This study did not involve field-collected samples

Ethics oversight

Animal protocols were approved by the Animal Care and Use Committee at Yale University

Note that full information on the approval of the study protocol must also be provided in the manuscript.

Human research participants

Policy information about studies involving human research participants

Population characteristics

Human scRNA seq data: Our analysis focused on 17 patients with advanced COPD, and 15 age-matched controls. Sample size for 10x Genomics scRNA-seq was determined by the availability of patient samples, as was the sample size for all other experiments. There were eight females in both groups and the median age of all subjects was 62 years old (range 41–80). All COPD subjects had radiographic evidence of advanced emphysema and were former smokers; four of the donors were either current or former smokers. Further details are highlighted in Supplemental Table 1. Using flow-sorted epithelial cells from single cell suspensions of 10 subjects with advanced COPD and 16 controls.

Recruitment

Human diseased and control explanted lungs were procured from donors with end-stage lung disease undergoing transplant or control lungs rejected for transplant, per protocols approved by the Partners IRB with informed consent. Findings should be generalizable to patients with advanced COPD requiring transplant but not to all COPD patients. Biases related to why control donor lungs were rejected from transplant may have impacted results.

Ethics oversight

Study protocols were approved by Partners Healthcare Institutional Board Review (IRB Protocol 2011P002419). informed consent was obtained from each subject.

Flow Cytometry

Plots

Confirm that:

- [x] The axis labels state the marker and fluorochrome used (e.g. CD4-FITC).
- [x] The axis scales are clearly visible. Include numbers along axes only for bottom left plot of group (a 'group' is an analysis of identical markers).
- [x] All plots are contour plots with outliers or pseudocolor plots.
- [x] A numerical value for number of cells or percentage (with statistics) is provided.

Methodology

Sample preparation

Small airway epithelial cells and A549 cells (American Type Culture Collection) were cultured in DMEM supplemented with 10% heat inactivated fetal bovine serum (FBS). The cells were passaged < 20 times. RNA duplexes for silencing NUPR1 (siNUPR1) and non-targeting control were obtained from Dharmacon. Cells were transfected in 12-well plates using RNAiMAX transfection reagent (Life Technologies) and OptiMEM media according to manufacturer's protocols. Cells were re-

	transfected after 72 hours and used for experimentation 24-48 hours after the second transfection, and after being incubated with 0% or 8% CSE for 20 hours. For CSE, mainstream smoke from one 3RF4 research cigarette (University of Kentucky, Lexington, Kentucky) was suctioned through 10 mL of cell culture media and filtered using a 0.22 µm filter (MilliporeSigma) and the obtained filtrate considered 100% CSE.
Instrument	Cytoflex LX flow cytometer
Software	FlowJo 10.6.2
Cell population abundance	50,000 events/sample were capture
Gating strategy	FSC/SSC was used to gate on cells following treatment with and without CSE in primary cells. Then Annexin V-FITC and propidium iodide gating was performed. Negative controls (Fluorescence minus one) in both CSE and no CSE groups and positive controls (higher CSE dosages) were used to identify positive and negative populations. Positive populations (evidence for cell death) were Annexin V + propidium iodide - or Annexin V + propidium iodide +. The negative population was defined as Annexin V- propidium iodide -.

☒ Tick this box to confirm that a figure exemplifying the gating strategy is provided in the Supplementary Information.
